# Cell death dependent on holins LrgAB repressed by a novel ArsR family regulator CdsR

**DOI:** 10.1038/s41420-024-01942-3

**Published:** 2024-04-11

**Authors:** Xin Zhang, Yuhan Chen, Tinglu Yan, Hengjie Wang, Ruibin Zhang, Yanrong Xu, Yujia Hou, Qi Peng, Fuping Song

**Affiliations:** 1 State Key Laboratory for Biology of Plant Diseases and Insect Pests, Institute of Plant Protection, Chinese Academy of Agricultural Sciences, Beijing, China; 2 College of Life Science, Northeast Agricultural University, Harbin, China

**Keywords:** Gene expression, Transcriptional regulatory elements, Gene regulation

## Abstract

The cell death and survival paradox in various biological processes requires clarification. While spore development causes maternal cell death in *Bacillus* species, the involvement of other cell death pathways in sporulation remains unknown. Here, we identified a novel ArsR family transcriptional regulator, CdsR, and found that the deletion of its encoding gene *cdsR* causes cell lysis and inhibits sporulation. To our knowledge, this is the first report of an ArsR family transcriptional regulator governing cell death. We found that CdsR directly repressed *lrgAB* expression. Furthermore, *lrgAB* overexpression resulted in cell lysis without sporulation, akin to the *cdsR* mutant, suggesting that LrgAB, a holin-like protein, induces cell death in *Bacillus* spp. The *lrgAB* mutation increases abnormal cell numbers during spore development. In conclusion, we propose that a novel repressor is vital for inhibiting LrgAB-dependent cell lysis.

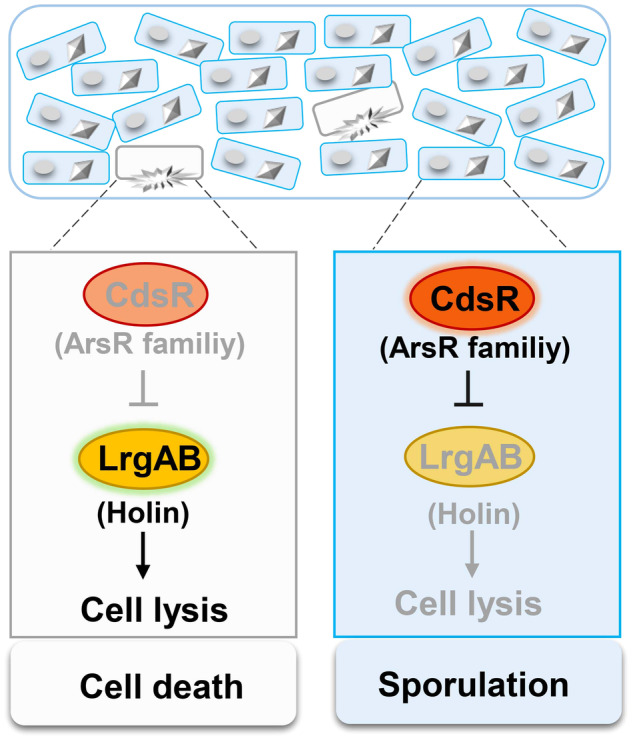

## Introduction

Cell death plays a key role in various biological processes. In eukaryotes, cell death encompasses apoptosis, necrosis, oncosis, pyroptosis, and autophagy [1]. These diverse forms of cell death have evolved to eliminate damaged and/or infected cells from affected tissues, enabling the surrounding healthy cells to function more effectively during development [2, 3]. In prokaryotes, cell death is an altruistic process that benefits the entire population [4]. Bacteria undergo altruistic death, conferring advantages on populations, such as vegetative growth [4], sporulation [5], biofilm maintenance [4], and bacteriophage-infection containment [6].

Cell death is reportedly involved in sporulation. Losick et al. proposed a model that elucidates how *Bacillus* populations delay sporulation through a cell death pathway [7, 8]. This model involves the impact of the killing factor SkfA-H and the spore delay protein SdpABC, which are associated with spore formation in *B. subtilis* under nutrient-limited conditions, causing the death of some cells within the population [7]. Nutrient limitation induces spore formation in *B. subtilis*, a process regulated by a gradual increase in the level and activity of the regulatory protein Spo0A [9]. Spo0A is activated by phosphorylation, a process facilitated by a multicomponent phosphoryl group delivery system [10]. This cascade starts with a relay protein, Spo0F, being phosphorylated by histidine protein kinases (KinA, KinB, and KinC) [11, 12]. Spo0F-P then transfers the phosphoryl group to Spo0B, which, in turn, transfers it to Spo0A [10, 13]. The resulting Spo0A~P can activate or repress the transcription of genes involved in sporulation, with its high thresholds activating the expression of *spoIIE*, *spoIIA*, and *spoIIG* [14–17], and low thresholds activating the expression of *skfA*-*H* and *sdpABC* [18]. Another mechanism that induces the cell death pathway in sporulating cells involves a quality control process that eliminates improperly sporulating cells individually [19]. In *B. subtilis*, the base layer of the sporulation coat contains a membrane-binding protein, SpoVM, which selectively localizes to the positively curved membrane surrounding the forespore [20]. SpoVM recruits the structural protein SpoIVA (ATPase derived from a family of GTPases), which utilizes the energy from ATP hydrolysis to drive its irreversible polymerization, forming a static platform as a foundation for the assembly of the remaining sporulation coat [19, 21]. CmpA acts as a molecular adaptor that interacts with SpoVM and SpoIVA, crucial proteins that scaffold the coat. This interaction aids in recruiting the protease ClpXP, which specifically degrades SpoIVA. Consequently, the degradation of SpoIVA triggers the lysis of sporulating cells. CmpA plays a role in quality control, overseeing the formation of the spore envelope and eliminates improperly developed spores [22]. This cell death pathway ensures the stable development of sporulating cells within the *B. subtilis* cell population during the late stages of sporulation. However, the genes related to cell lysis that maintain the fidelity of sporulation remain unknown.

The Cid/Lrg system-mediated bacterial death and lysis occur in the context of developing biofilms in *Staphylococcus aureus* and represent a well-recognized cell death pathway [23, 24]. The *cidABC* and *lrgAB* operons regulate autolysis and extracellular DNA release by controlling peptidoglycan hydrolase activity via holin- and antiholin-like proteins such as CidA and LrgA [24, 25]. However, a recent report concluded that *lrgA* encodes proteins with bacteriophage holin-like functions, contributing to cell death in *S. aureus* [26]. Holins are a class of small membrane proteins required for the phage lysis of a host cell, enabling the formation of a membrane hole. Murein hydrolases in the cytoplasm can then escape and degrade peptidoglycans, ultimately reducing the membrane potential and causing cell death [27]. Homologs of *lrgAB* are widely distributed in bacteria, including both gram-negative and gram-positive bacteria. However, the role of LrgAB in cell lysis, particularly in biological processes like sporulation, still requires further elucidation, besides its established function in *S. aureus*.

*Bacillus thuringiensis* is a crucial biocontrol agent widely employed for agricultural pest management, owing to its ability to produce a protein-like crystalline toxin (δ-endotoxin) with specific activity against the gut of certain insect larvae [28]. Interestingly, *B. thuringiensis* shares a highly similar sporulation program with *B. subtilis* [29, 30], making it an ideal model for exploring the relationship between cell death and sporulation. In this study, we identified the ArsR family regulatory gene *cdsR* (*HD73_RS03760*, cell death and sporulation regulator) involved in cell death in *B. thuringiensis* subsp. *kurstaki* HD73 (hereafter referred to as HD73). Deletion of the *cdsR* gene resulted in a significant autolytic phenotype during liquid cultivation, with CdsR acting as a transcriptional repressor of the *lrgAB* operon. Overexpressing *lrgAB* triggered cell lysis and inhibited sporulation in wild-type HD73 cells. Deleting *lrgAB* resulted in an increased number of abnormal cells. These findings revealed, for the first time to our knowledge, that a novel repressor plays a crucial role in inhibiting cell autolysis mediated by the LrgAB holin-like system.

## Results

### Mutation of *cdsR* results in cell lysis and no sporulation

**Fig. 1:**
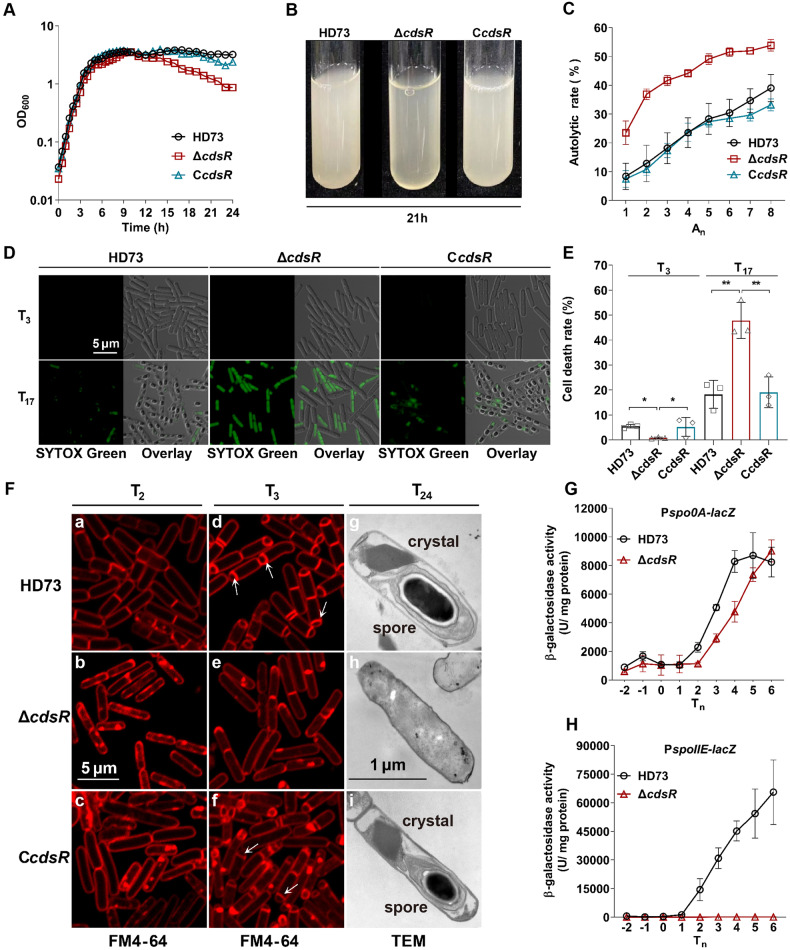
Identification of the CdsR, a suppressor that controls the cell death. **A** Role of CdsR in the growth of *Bacillus thuringiensis* HD73. Growth curves of HD73 (black circles), Δ*cdsR* (red squares), and C*cdsR* (blue triangles) cultured in Schaeffer’s sporulation medium (SSM) at 30 °C with shaking at 220 rpm were plotted by measuring the optical density at 600 nm (OD_600_) against incubation time. **B** Autolysis images of HD73, Δ*cdsR*, and C*cdsR* in SSM at 21 h. Samples (5 mL) were collected from a 500 mL triangular bottle and photographed in clear glass test tubes. **C** The autolytic rate, expressed as the percentage increase in the OD_580_, was determined for hourly samples up to T_6_. Mean values (SD, *n* = 3) are represented by symbols: HD73 (black circles), Δ*cdsR* (red squares), and C*cdsR* (blue triangles). **D** Imaging of cell death in *B. thuringiensis* cell populations using SYTOX^TM^ Green at T_3_ and T_17_ for HD73 and Δ*cdsR*, with a scale bar of 5 μm. **E** Statistical analysis of cell death rate in *B. thuringiensis* cells, calculated by comparing the number of dead cells to the total cell count in photographs. The cell death rate is expressed as the ratio of the number of cells exhibiting green fluorescence to the number of total cells, multiplied by 100. Values display the means of at least three independent replicates. The cell death rates of Δ*cdsR* and C*cdsR* were compared with HD73 and analyzed using a T-test (*P < 0.05; **P < 0.01). **F** Polar septum formation during spore initiation was observed using laser confocal microscopy. Strains grown to T_2_ and T_3_ represent HD73, Δ*cdsR*, and C*cdsR*, with red lines indicating membranes stained with FM4-64. Transmission electron microscopic (TEM) observation was conducted to determine the cell shape and sporulation structure of HD73, Δ*cdsR*, and C*cdsR* strains at T_24_. **G** Construction of a transcriptional reporter by fusing the *spo0A* gene promoter with *lacZ*. The β-galactosidase activities of three clones were determined at specified time points following cell growth in SSM at 30 °C. Each value represents the mean and standard error of at least three independent replicates. **H** Fusion of the *spoIIE* gene promoter with *lacZ* to construct a transcriptional reporter.

Numerous unidentified regulators are present in *B. thuringiensis* HD73. To elucidate their functions, we analyzed the expression levels of regulator-encoding genes during the stationary phase using our previous DNA microarray data (Accession No. GSE 48410) [31]. We selected thirty candidate genes with high relative expression levels (Table S1) and their mutants were generated through homologs recombination. Among these mutants, the Δ*HD73_RS03760* mutant exhibited autolysis and inability to form endospores (Fig. 1). Therefore, it was designated as *cdsR* (cell death and sporulation regulator, *cdsR*).

To determine the effect of *cdsR* mutation on cell growth, we plotted a growth curve, which showed a significant reduction in the cell density of Δ*cdsR* from 14 to 24 h compared to that of the wild-type HD73 strain (Fig. 1A). However, the cell density was restored in the genetically complementary strain C*cdsR* (Fig. 1A). In a test tube, the OD_600_ values of HD73, Δ*cdsR*, and C*cdsR* were 3.166, 1.226, and 2.699 at 21 h, respectively (Fig. 1B). This suggests that *cdsR* deletion causes autolysis during the late stationary phase. To determine the autolytic rate of the Δ*cdsR* and HD73, we collected cells at T_6_, induced autolysis with Triton X-100, and measured the OD_580_ value every hour. The results showed that the autolytic rate of Δ*cdsR* mutant was significantly higher than that of HD73 and C*cdsR* from A_1_ to A_8_ (Fig. 1C). These results strongly indicate that CdsR acts as a repressor of cell lysis in HD73 cells.

To determine the effect of *cdsR* on cell death, cells were stained with a sensitive nucleic acid dye, SYTOX^TM^ Green, to visualize dead cells using laser confocal microscopy. The cell death rate was calculated as the ratio of the number of dead cells (stained with SYTOX^TM^ Green) to the total number of cells. Fewer cells with green fluorescence were observed in HD73 or Δ*cdsR* at T_3_, but they became more apparent at T_17_ (Fig. 1D). At T_17_, 47.85(±6.45)% of Δ*cdsR* cells were dead, whereas only 18.22(±5.00)% and 19.09(±5.53)% were dead in HD73 and C*cdsR*, respectively (Fig. 1E). This indicated that the deletion of the *cdsR* gene increases cell death.

It is noteworthy that the Δ*cdsR* strain did not produce spores and parasporal crystals, in contrast to the wild-type HD73 and C*cdsR* (Fig. 1Fg, Fi). To elucidate the reason for the absence of spores in Δ*cdsR*, the formation of a polar septum during sporulation was observed by laser confocal microscopy. The cell membranes of HD73, Δ*cdsR*, and C*cdsR* cells were stained with FM4-64, which labels the plasma membranes of living cells. At T_2_ in SSM, vegetative cells were observed in HD73 (Fig. 1Fa), Δ*cdsR* (Fig. 1Fb), and C*cdsR* (Fig. 1Fc). At T_3_ in SSM, the polar septum was observed in the HD73 (Fig. 1Fd) and C*cdsR* (Fig. 1Ff), while Δ*cdsR* (Fig. 1Fe) showed no polar septum formation at all. Furthermore, forespore engulfment was visualized through staining with FM4-64 and MitoTracker Green FM (MTG). HD73 and C*cdsR* cells completed the engulfment process by T_7_ (Fig. S1b and S1h). In contrast, Δ*cdsR* cells still lacked a polar septum at T_7_ (Fig. S1e). This suggests that the effect of *cdsR* deletion on sporulation occurs before polar septum formation.

Spo0A is the master regulator of sporulation initiation in *Bacillus* [9, 32]. To determine whether *cdsR* affects the transcription of Spo0A, the transcriptional activity of the *spo0A* promoter was analyzed in HD73 and Δ*cdsR* strains. The β-galactosidase assay showed no significant difference in the transcriptional activity of *spo0A* between HD73 and Δ*cdsR* (Fig. 1G). The activity of Spo0A is determined by its phosphorylation level, and the phosphorylated Spo0A protein can activate the transcription of *spoIIE* gene in *B. subtilis* [15] and *B. thuringiensis* [29, 33]. Therefore, the promoter activity of *spoIIE* can serve as an indicator of the activity of Spo0A [32]. The β-galactosidase assay showed high transcriptional activity of *spoIIE* in HD73 from T_2_ to T_6_, whereas no transcriptional activity of *spoIIE* was detected in Δ*cdsR* (Fig. 1H). This suggests that CdsR modulates sporulation by affecting Spo0A activity.

### CdsR is negatively autoregulated

**Fig. 2:**
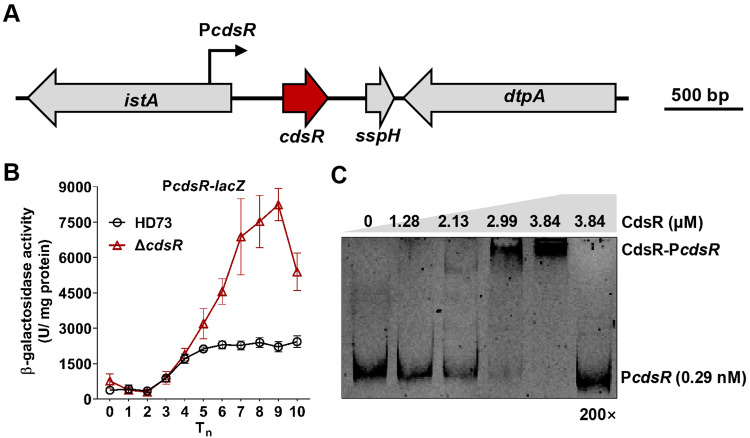
CdsR is a novel small regulator exhibiting negative autoregulation. **A** Genetic loci of the *cdsR* gene in HD73. In the genome region of the *cdsR* gene (encoding cell death and survival repressor, indicated by the red arrow), the upstream and downstream genes are *HD73_RS03755* (encoding IS21-like element IS232 family transposase) and *HD73_RS03765* (encoding acid-soluble spore protein H), and *HD73_RS03770* (encoding peptide MFS transporter), respectively. The black arrow represents the *cdsR* promoter region. **B** Transcription analysis of the *cdsR* promoter in HD73 and Δ*cdsR*. The β-galactosidase activities of three clones were determined at specified time points after cultivating *B. thuringiensis* cells in SSM at 30 °C. Each value represents the mean and standard error of at least three independent replicates. **C** Detection of the interaction between the *cdsR* promoter and increasing concentrations of the CdsR protein through electrophoretic mobility shift assay (EMSA). The lanes contained 0, 1.28, 2.13, 2.99, and 3.84 μM of the CdsR protein. The final lane contained 200-fold unlabeled probes to compete with the combined FAM-labeled probes.

CdsR consists of 95 amino acids and belongs to the ArsR family of transcriptional regulators, which include a single helix-turn-helix (HTH) DNA-binding domain ranging from 15 to 90 amino acid residues. The genes upstream and downstream of *cdsR* include *HD73_RS03755* (*istA*), *HD73_RS03765* (*sspH*), and *HD73_RS03770* (*dtpA*), which encode the IS21-like element IS232 family transposase, acid-soluble spore protein H, and peptide MFS transporter, respectively (Fig. 2A). To determine the transcriptional levels of the *cdsR* gene, the *cdsR* promoter (P*cdsR*) was fused with the *lacZ* reporter gene, and the activity of P*cdsR* in HD73 and Δ*cdsR* were measured. The β-galactosidase assay showed that in HD73, the activity of the P*cdsR* remained low from T_0_ to T_2_, increased significantly from T_2_ to T_5,_ and remained high from T_5_ to T_10_ (Fig. 2B). However, in Δ*cdsR*, the transcriptional activity of P*cdsR* was significantly higher than in HD73 from T_4_ to T_10_ (Fig. 2B). To confirm whether the CdsR protein could bind to its own promoter, it was expressed and purified in *E. coli* (Fig. S2), and an electrophoretic mobility shift assay (EMSA) was conducted. Various concentrations of the CdsR protein were used to bind to 0.29 nM of the P*cdsR*-labeled probe. At low concentrations (1.28 μM and 2.13 μM), the CdsR protein was unable to effectively bind to the P*cdsR*-labeled probe. However, at a higher concentration of 3.84 μM, CdsR was able to bind and completely shift 0.29 nM of P*cdsR*-labeled probe. Notably, a 200-fold excess of unlabeled probe competed with the labeled probe, confirming specific binding (Fig. 2C). These results collectively indicate that CdsR undergoes negative autoregulation.

### CdsR directly regulates *lrgAB* and *cwlD* expression

**Fig. 3:**
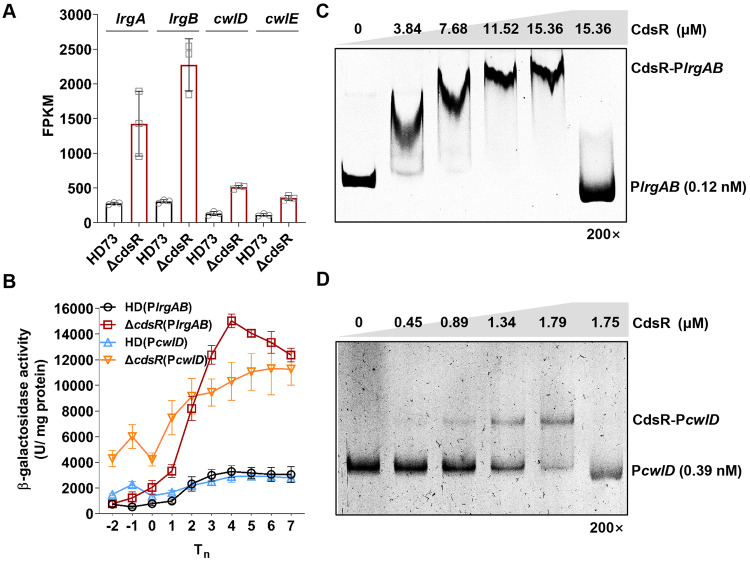
CdsR negatively regulates the expression of the *lrgAB* operon. **A** Analysis of the Δ*cdsR* transcriptome at T_0_. The differentially expressed genes associated with cell autolysis (*lrgA*, *lrgB*, *cwlD*, and *cwlE*) were analyzed according to fragments per kilobase of transcript per million mapped reads (FPKM). **B** Transcriptional analysis of the promoter regions of autolysis-related genes (*lrgAB* and *cwlD*) fused to *lacZ* in HD73 and Δ*cdsR*, respectively. Black circles indicate the HD(P*lrgAB*) strain, red squares indicate the Δ*cdsR*(P*lrgAB*) strain, blue triangles indicate the HD(P*cwlD*) strain, and yellow inverted triangles indicate the Δ*cdsR*(P*cwlD*) strain. **C** Examination of the interaction between the CdsR and P*lrgAB* promoter regions via electrophoretic mobility shift assay (EMSA), with increasing quantities of purified CdsR protein incubated with the P*lrgAB* promoter regions. The last lane was employed for the 200-fold unlabeled probe. **D** Increasing amounts of purified CdsR protein incubated with the P*cwlD* promoter regions. The last lane was used for the 200-fold unlabeled probe.

Previous findings showed that the *cdsR* gene was transcribed at a low level (Fig. 2B) and had an impact on the onset of the stationary phase (Fig. 1H). To identify potential autolysis and sporulation-related target genes of CdsR, the transcriptomes of HD73 and Δ*cdsR* at T_0_ were compared. Differentially expressed genes were screened based on a fold-change>2 and a false discovery rate cutoff of *P* < 0.05, using RNA sequencing (RNA-Seq) data. The RNA-Seq data were deposited in the NCBI Gene Expression Omnibus database (accession no. GSE216307). The analysis revealed 728 genes with differential expression in Δ*cdsR* relative to the HD73. Among these genes, 514 were upregulated, and 214 were downregulated. To investigate the functional roles of these differentially expressed genes, they were classified into groups related to amino acid metabolism, signaling, cellular processing, and virulence, according to the KEGG function (Table S2). While CdsR was shown to modulate sporulation primarily by affecting Spo0A activity (Fig. 1G, H), the RNA-Seq data showed that the transcription of genes involved in Spo0A activity (such as *kinA*-*E*, *spo0F*, *spo0B*, *phr*-*rap*, and *spo0E,* etc.) exhibited no significant change between HD73 and Δ*cdsR* (Table S3). Notably, RNA-Seq analysis identified only four upregulated genes associated with autolysis, including *HD73_RS29150* (*lrgA*, encoding a holin-like protein), *HD73_RS29145* (*lrgB*, encoding an antiholin-like protein; *lrgA* and *lrgB* compose the *lrgAB* operon), *HD73_RS20125* (encoding N-acetylmuramoyl-L-alanine amidase, named *cwlD*), and *HD73_RS04330* (encoding N-acetylmuramoyl-L-alanine amidase, named *cwlE*). The RNA-Seq data revealed that the FPKM values of *lrgA* and *lrgB* were higher than those of the *cwlD* and *cwlE* genes in Δ*cdsR* at T_0_ (Fig. 3A).

To detect the transcription dynamics of these genes, the *lrgAB* (P*lrgAB*), *cwlD* (P*cwlD*), and *cwlE* promoters (P*cwlE*) were fused to *lacZ* reporter gene. Subsequently, we investigated the expression of these three genes/operons in both HD73 and Δ*cdsR* through β-galactosidase assays. The result showed that the activities of the P*lrgAB* and P*cwlD* were significantly higher in Δ*cdsR* than in HD73. Notably, the transcriptional activities of P*lrgAB* and P*cwlD* were similar in HD73 cells (Fig. 3B). However, P*cwlE* activity was extremely low in both HD73 and Δ*cdsR* mutant (Fig. S3). These results clearly indicate that CdsR represses the transcription of *lrgAB* and *cwlD*.

To determine whether CdsR binds to the promoter regions of *lrgAB*, *cwlD*, and *cwlE*, we conducted an EMSA. Various concentrations of the CdsR protein were used to bind to 0.12 nM of the P*lrgAB*-labeled probe. Notably, low concentrations of the CdsR protein (3.84 and 7.68 μM) were able to effectively bind to the P*lrgAB*-labeled probe. The P*lrgAB*-labeled probe at a concentration of 0.12 nM was completely shifted with 15.36 μM of CdsR. However, a 200-fold excess of unlabeled probe successfully competed with the labeled probe, indicating specific binding (Fig. 3C). In another EMSA, 1.75 μM of CdsR was used to bind to 0.39 nM P*cwlD*-labeled probe. Remarkably, low concentrations of CdsR protein (0.89 and 1.34 μM) were able to effectively bind to the P*cwlD*-labeled probe. The P*cwlD*-labeled probe at a concentration of 0.39 nM was completely shifted with 1.75 μM of CdsR. A 200-fold excess of unlabeled probe successfully competed with the labeled probe, confirming specific binding (Fig. 3D). However, when 0.3 nM of the P*cwlE*-labeled probe was exposed to CdsR concentration ranging from 1.49 μM to 5.95 μM, no binding occurred, indicating that CdsR cannot interact with the *cwlE* promoter region (Fig. S3). These results strongly support the direct repression of *lrgAB* and *cwlD* by CdsR.

### Overexpression of *lrgAB* results in cell lysis without sporulation

Holins act in conjunction with endolysins to cause cell lysis [34, 35]. Although *lrgAB* was initially annotated as an antiholin protein, a recent study confirmed that LrgAB can indeed act as a holin, inducing cell lysis in *S. aureus* [26]. The heightened transcriptional activity of *lrgAB* and *cwlD* in Δ*cdsR* in comparison to HD73 suggests that LrgAB, acting as a holin, and CwlD, serving as an endolysin, may collaborate to induce cell lysis. However, it is imperative to ascertain the impact of the overexpression of the *lrgAB* operon, *lrgA*, or *lrgB* on cell lysis.

**Fig. 4:**
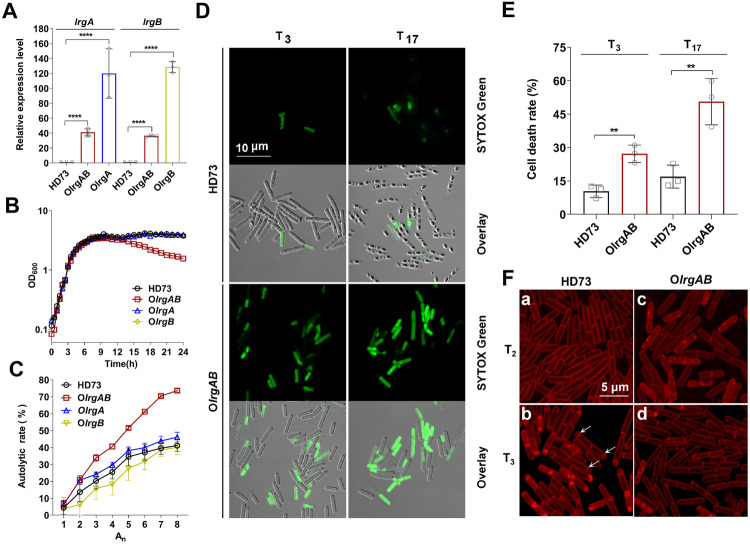
Overexpression of LrgAB results in cell lysis without sporulation. **A** Analysis of *lrgA* expression in HD73, O*lrgAB,* and O*lrgA* strains and *lrgB* expression in HD73, O*lrgAB,* and *OlrgB* strains by RT-qPCR at T_5_. Experiments were conducted in triplicate. Error bars represent standard deviations (****P < 0.0001). **B** Role of LrgAB in the growth of *B. thuringiensis*. Growth curves were plotted for HD73 (black circles), O*lrgAB* (red squares), O*lrgA* (blue triangles), and O*lrgB* (yellow diamond) strains grown in SSM at 30 °C with shaking at 220 rpm. **C** Autolytic rates of HD73, O*lrgAB*, O*lrgA*, and O*lrgB* in Tris-HCl and Triton X-100 mixed buffer, expressed as a percentage increase in OD_*580*_. **D** Cell death imaging in *B. thuringiensis* cell populations using SYTOX^TM^ Green at T_3_ and T_17_ for HD73 and O*lrgAB*. Bar, 10 μm. **E** Statistical analysis of the cell death rates of the HD73 and O*lrgAB* strains, obtained by counting the number of dead and live cells from microscopic images. The cell death rate of O*lrgAB* was compared to that of HD73, and the data were analyzed using a T-test (**P < 0.01). **F** Observation of polar septum formation during spore initiation by laser confocal microscopy. The strains grown in SSM at T_2_ and T_3_ correspond to HD73 and O*lrgAB*, respectively.

O*lrgAB*, O*lrgA,* and O*lrgB* strains were generated by employing the pHT304 vector to overexpress the *lrgAB* operon, *lrgA*, and *lrgB* as described in “Materials and methods” section. An RT-qPCR analysis revealed that the transcriptional level of *lrgA* increased by approximately 100- and 30-fold in O*lrgA* and O*lrgAB* strains, respectively, compared to HD73. Similarly, *lrgB* exhibited increased transcription in both O*lrgB* and O*lrgAB* strains compared to HD73 cells (Fig. 4A), unequivocally confirming the successful overexpression of *lrgA* and *lrgB* in the corresponding strains.

The growth curve showed a significant reduction in cell density for O*lrgAB* from 14 to 24 h compared to the HD73, O*lrgA*, and O*lrgB* strains (Fig. 4B). Similarly, the autolytic rate of O*lrgAB* was significantly higher than that of HD73, O*lrgA*, and O*lrgB* from A_1_ to A_8_ (Fig. 4C), indicating an increased autolytic activity in the O*lrgAB* strain. Taken together, the simultaneous expression of *lrgA* and *lrgB* led to cellular autolysis, a phenomenon that did not occur when *lrgA* and *lrgB* were overexpressed alone. These findings suggest that LrgAB is a critical complex responsible for cell lysis in HD73. The proportion of dead cells was determined using SYTOX^TM^ Green staining, with fewer cells exhibiting green fluorescence in HD73 cells compared to O*lrgAB* cells at both T_3_ and T_17_ (Fig. 4D). A quantitative analysis revealed that the proportion of dead cells was 16.93(±4.63)% in HD73 and 50.63(±9.31)% in O*lrgAB* at T_17_ (Fig. 4E). Collectively, these results indicate that the overexpression of *lrgAB* induces cell lysis.

When HD73 and O*lrgAB* cells cultured in SSM were stained with FM4-64, polar septa were observed in HD73 cells (Figs. 4Fa and 4Fb), whereas O*lrgAB* cells exhibited no polar septa at T_3_ (Fig. 4Fd). In additon, HD73 cells completed the process of engulfment at T_7_, whereas O*lrgAB* cells showed no polar septum formation (Fig. S1k). In summary, O*lrgAB* and Δ*cdsR* exhibit similar phenotypes, characterized by cell lysis and the absence of sporulation (Figs. 1 and 4). This indicates that CdsR plays a critical role in cell lysis and sporulation by regulating *lrgAB*.

### Deletion of *lrgAB* leads to a greater number of abnormal cells

**Fig. 5:**
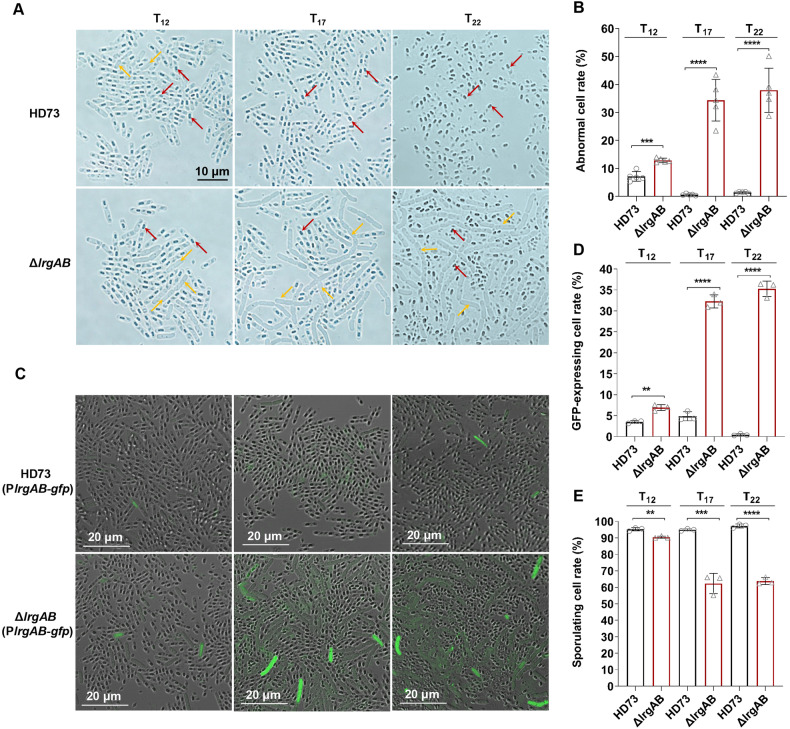
*lrgAB* deletion mutant enhances abnormal cell number. **A** Morphological modification of Δ*lrgAB* by optical microscopy. Bar, 10 μm. Red arrows indicate spore cells, and yellow arrows indicate abnormal cells. **B** Statistical analysis of the abnormal cell rates of HD73 and Δ*lrgAB*, obtained by counting the number of sporulating and abnormal cells from microscopic images. The abnormal cell rate of Δ*lrgAB* was compared to that of HD73, and the data were analyzed using the T-test (***P < 0.001. ****P < 0.0001). **C** Laser confocal microscopy was performed to determine the expression of *lrgAB* in Δ*lrgAB*. P*lrgAB* promoter-directed *gfp* gene expression was examined in Δ*lrgAB* and HD73. Bar, 20 μm. **D** The GFP-expressing cell rate of the Δ*lrgAB* was compared to that of HD73. The percentage of GFP-expressing cells in HD73 and Δ*lrgAB* was statistically analyzed by counting the number of total cells and GFP-expressing cells through micrographs. **E** The sporulating cell rate of the Δ*lrgAB* was compared with that of HD73. The percentage of sporulating cells in Δ*lrgAB* and HD73 was statistically analyzed by counting the number of total cells and sporulating cells through micrographs. Data were obtained from three random microscopy images and analyzed by T-test (**P < 0.01. ***P < 0.001. ****P < 0.0001).

We demonstrated that LrgAB induces cell lysis without sporulation, and this regulation is mediated by CdsR. This highlights the essential role of the cell death pathway regulated by LrgAB in the sporulation process and emphasizes its tight regulation. Further investigation into the effects of disrupting this pathway on sporulation would be highly intriguing. Optical microscopy revealed the presence of two distinct cell types: sporulating cells and abnormal cells lacking spore formation ability, displaying a deformed cell shape in both HD73 and the Δ*lrgAB* mutant (Fig. 5A). A quantitative analysis revealed that the proportions of abnormal cells in the total cell population at T_12_, T_17_, and T_22_ were 7.13(± 1.72)%, 0.61(± 0.28)%, and 1.38(± 0.3)%, respectively, in HD73, and 12.87(± 0.75)%, 34.35(± 7.43)%, and 37.94(± 7.86)%, respectively, in the Δ*lrgAB* mutant (Fig. 5B). The Δ*lrgAB* mutant exhibited a significant accumulation of abnormal cells compared to HD73. These findings suggest that *lrgAB* deletion leads to a greater number of abnormal cells.

To clarify *lrgAB* expression in abnormal cells, a fusion expression vector harboring the P*lrgAB* promoter and the *gfp* gene was introduced into HD73 and Δ*lrgAB* mutant cells, respectively. Laser confocal microscopy revealed that the number of cells exhibiting green fluorescence at T_12_, T_17_, and T_22_ remained extremely low and did not accumulate in HD73 cells over time. In contrast, while very few cells exhibited green fluorescent at T_12_, a higher number of non-sporulating cells expressing green fluorescent protein appeared in the Δ*lrgAB* mutant at T_17_ and T_22_ (Fig. 5C). Quantification of cells showed that in HD73, GFP-expressing cells accounted for 3.50(± 0.31)%, 4.84(± 1.07)%, and 0.44(± 0.22)% of the total cells at T_12_, T_17_, and T_22_, respectively. However, in the Δ*lrgAB* mutant, GFP-expressing cells represented 6.92(± 0.71)%, 32.27(± 1.55)%, and 35.24(± 1.84)% of the total cells at T_12_, T_17_, and T_22_, respectively (Fig. 5D). This suggests that LrgAB is activated in abnormal cells.

To determine the role of the LrgAB system in sporulation, the number of total cells and sporulating cells were quantified. The result of the plate count showed that the total number of HD73 (1.358 × 10^7^ CFU/mL) and Δ*lrgAB* (1.352 × 10^7^ CFU/mL) cells were similar (Fig. S4). However, the result of quantification through microscopy images showed that the sporulating cell rate of HD73 (95.23(± 1.03)%, 94.92(± 0.62)%, and 97.26(± 1.17)%) was higher than that of Δ*lrgAB* (90.42(± 0.64)%, 62.29(± 6.12)%, and 63.78(± 2.14)%) at T_12_, T_17_, and T_22_, respectively (Fig. 5E). This suggests that the LrgAB system plays a crucial role in the sporulating cell population. In summary, deletion of *lrgAB* leads to an accumulation of abnormal cells and the P*lrgAB* promoter is more active in abnormal cells.

## Discussion

In this study, we identified a novel transcriptional regulator, CdsR, which inhibits cell lysis and promotes sporulation in *B. thuringiensis* (Fig. 1). We analyzed CdsR homologs with >30% similarity and >60% coverage. We examined 75 sequences from annotated and publicly available bacterial genomes that represent species diversity in terms of evolutionary distance using the Basic Local Alignment Search Tool BLASTP (https://blast.ncbi.nlm.nih.gov/Blast.cgi?PAGE=Proteins). Subsequently, the sequences corresponding to the best hits were subjected to multiple sequence alignment using ClustalW (https://www.genome.jp/tools-bin/clustalw). Thereafter, we constructed phylogenetic trees based on the alignments and found that CdsR homologs are present in a variety of bacterial species. Further analysis revealed that CdsR homologs were primarily distributed in the *B. cereus* group, *Clostridium* spp., *Paenibacillus* spp., and *Selenomonas* spp. (Fig. S5). CdsR homologs with 100% similarity were predominantly found in the *B. cereus* group, which included *B. thuringiensis* known for its insecticidal properties, *B. cereus* associated with emetic and/or diarrheal foodborne illnesses as food poisoning agents, and *B. anthracis* responsible for anthrax [36, 37]. This suggests that the mechanisms underlying CdsR-dependent inhibition of cell lysis and promotion of sporulation might be similar within the *B. cereus* group. CdsR belongs to the ArsR family of regulators, which are known to be involved in various functions such as metal ion homeostasis [38], biofilm formation [39], and virulence [40]. Generally, the N-terminus of ArsR family regulators contains a winged helix-turn-helix DNA-binding domain that represses transcription by binding to downstream target promoters [41]. In this study, we found that a novel ArsR family regulator plays an important role in cell death and sporulation.

Homologs of *lrgAB* are found across a wide range of bacteria, including gram-negative bacteria (*Fusobacterium* spp., *Bradyrhizobium* spp., *Brucella* spp., *Rhodobacter* spp., *Burkholderia* spp., *Chromobacterium* spp., *Desulfuromonas* spp., *Escherichia* spp., *Salmonella* spp., and *Yersinia* spp.), gram-positive bacteria (*Kineococcus* spp., *Streptomyces* spp., *Deinococcus* spp., *Bacillus* spp., *Exiguobacterium* spp., *Staphylococcus* spp., *Clostridium* spp., *Enterococcus faecalis*, *Pediococcus* spp., *Lactococcus* spp., and *Streptococcus* spp.), and archaea (*Methanosarcina* spp. and *Pyrococcus* spp.) [24]. These LrgAB homologs were initially annotated as antiholin-like proteins until the discovery of LrgA in *S. aureus*, which was identified as a bacteriophage holin-like protein that plays a role in cell death [26]. In *Bacillus* spp., LrgAB proteins have various functions across different strains. For instance, the *lrgA* homolog, *ysbA*, is not involved in cell lysis but is essential for pyruvate transport or utilization in *B. subtilis* [42]. In *B. cereus*, LrgAB is involved in extracellular DNA release and biofilm formation [43]. Mutations in *lrgAB* affect stationary-phase survival and sporulation efficiency in *B. anthracis* [5]. A comparison of the amino acid sequences of available LrgA homologs revealed that LrgA (HD73_RS29150) of *B. thuringiensis* HD73 shares 62%, 99%, and 100% similarity with LrgA (YsbA, BSU28910) of *B. subtilis* 168, LrgA (BC5439) of *B. cereus* ATCC 14579, and LrgA (BA5690) of *B. anthracis* Ames, respectively. In our previous study, we found that deletion of *lrgAB* did not significantly affect sporulation efficiency by the method of heating cells for elimination of non-sporulating cells [44]. In this study, we directly counted the number of sporulating cells in the microscopy images. We found that the total number of cells in HD73 and Δ*lrgAB* mutant was comparable (Fig. S4), but the rate of sporulating cell in HD73 was significantly higher than that in Δ*lrgAB* mutant (Fig. 5E). The difference may be due to the heating method in which some non-sporulating cell also showed thermal resistance. The aforementioned studies on *Bacillus* species have predominantly focused on the effects of deleting *lrgAB*. However, in this study, we found that in *B. thuringiensis*, the overexpression of LrgAB plays a role in both cell death and sporulation, functioning as a holin-like protein (Fig. 4). Notably, only the O*lrgAB* strain showed cell lysis, whereas the O*lrgA*, O*lrgB* and HD73 strains showed no cell lysis (Fig. 4). A previous study by Bayles and Endres suggested that LrgA alone can oligomerize and form pores within the cytoplasmic membrane, leading to cell lysis. However, our findings indicate that these functions must be performed in the context of LrgB [26]. Therefore, we speculate that *lrgAB* overexpression disrupts the cell membrane structure, and that *lrgA* and *lrgB* expressed alone are incapable of performing such biological functions in *B. thuringiensis*. To our knowledge, this is the first report on LrgAB acting as a holin-like protein that induces cell death in *Bacillus* spp.

Within the *B. cereus* group, there are three additional homologs of *lrgA*, namely *cidA*, *clhA1*, and *clhA2* [5, 45]. While disrupting of *B. anthracis clhAB2* had a minor impact on murein hydrolase activity, it was found to be crucial for sporulation [5]. However, the molecular mechanisms underlying the roles of *cidAB* and *clhAB1* (not transcription) in *B. anthracis* cell death remain unclear [5]. The amino acid sequence similarity of homologs CidA (HD73_RS19705), ClhA1 (HD73_RS20090), and ClhA2 (HD73_RS27290) comparisons with LrgA (HD73_RS29150) were 24% for CidA, 29% for ClhA1, and 26% for ClhA2 in *B. thuringiensis*, respectively. Among the four *lrgA* homologs in the HD73 strain, only *lrgA* exhibited upregulated expression with a 2.36-fold in Δ*cdsR*, and other three genes had no change (Table S5). This suggests that CdsR is involved in cell lysis and sporulation via regulation of the *lrgAB* operon. CdsR inhibits *lrgAB* expression via the cell death pathway. Although activation of *lrgAB* by LytSR and CidR positive regulation of *lrgAB* expression in *B. anthracis* [5] and *S. aureus* [24] has been well studied, very few repression regulators have been reported. *lrgAB* is negatively regulated by CcpA in *B. subtilis* and *Streptococcus mutans* during the exponential phase [46]. We report, for the first time, that CdsR represses LrgAB during the stationary phase.

Cell death can help eliminate unwanted or damaged cells and maintain the health of a population [47–49]. For example, cell death as a critical player in establishing a properly formed central nervous system by removing genetically compromised cells. The AIM2 inflammasome monitors DNA damage accumulation in neurons through its regulation of Gasdermin-D-mediated cell death, contributing to the elimination of genetically compromised central nervous system cells [47]. Physiological apoptosis is mediated by VAB-1/EphR receptor signaling, which promotes apoptosis in the *Caenorhabditis elegans* germline. In *C. elegans*, as germ cells progress through meiosis, about half of them undergo physiological apoptosis, in which their cytosolic components are redistributed to ‘nurse’ the remaining oocytes [48]. Furthermore, cell death is activated in response to DNA damage in a subset of *Pseudomonas aeruginosa* cells [49]. DNA damage results in the derepression of *alpA* expression, leading to the cleavage of genes, such as *alpBCDE*, which are activated for expression [50]. Thus, some lysed *P. aeruginosa* cells provide nutrients or liberate toxins or other bacterial factors that promote colonization of the lung by the remaining cells in the population [49]. In this study, the absence of *lrgAB* resulted in the inability to remove abnormal cells, which gradually accumulated in the *B. thuringiensis* population (Fig. 5). This is most likely a new example of a cell death pathway increases the benefits of a cell population.

In previous studies, a series of quality control systems have been identified to maintain the fidelity of sporulation program [19, 22, 51]. Sda, a suppressor of DnaA, and SirA, a sporulation inhibitor of replication A, control cells to enter sporulation in response to DNA replication defects. DisA is a DNA integrity scanning protein that functions as part of a DNA damage checkpoint immediately following the initiation of sporulation [21]. CmpA persists and acts as an adaptor of ClpXP to mediate degradation of SpoIVA in a σ^K^-dependent manner, and SpoVID monitors the polymerization state of the coat basement layer via an extension to a functional intracellular LysM domain [52], which eradicate sporulating cells that inaccurately assemble the envelope through cell lysis. However, the checkpoint-mediated cell death pathway in *B. subtilis* remains unclear. In this study, we found that LrgAB acts as a holin-like protein and that LrgAB-dependent cell death pathways maybe increase the benefit of sporulating cells (Fig. 5). It is one possibility that this cell death pathway followed the checkpoint system result in cell lysis. Therefore, the relationship between quality control systems and LrgAB-dependent cell death requires further investigation.

**Fig. 6:**
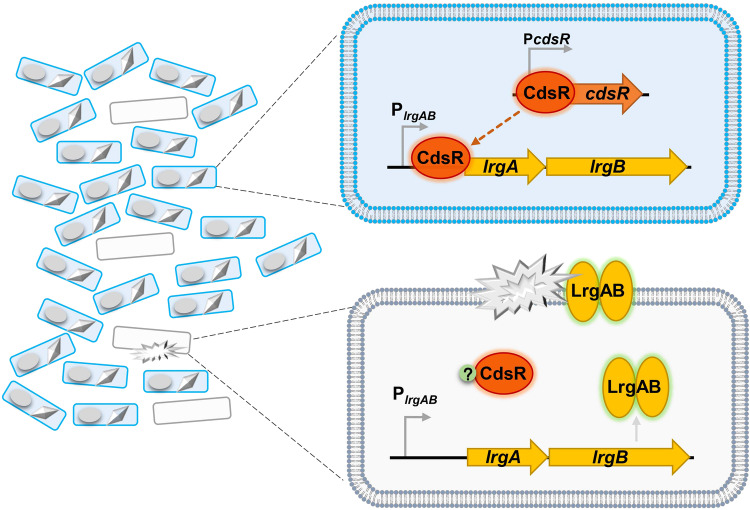
CdsR-mediated negative regulation in sporulating cell populations. Left: The HD73 cell population comprises two types of cells: sporulating cells (fully developed) and abnormal cells (abnormally developed). Right (top): CdsR serves as an autoregulator that negatively regulates *lrgAB* expression in sporulating cells. Right (bottom): In abnormal cells, CdsR relieves its inhibition of *lrgAB* through an unknown signal. LrgAB acts as a holin that induces cell lysis. Therefore, the CdsR-LrgAB cell death system is beneficial for the sporulation.

In summary, we propose a model for the LrgAB-dependent cell death pathway, which is repressed by the novel regulator, CdsR (Fig. 6). CdsR is negatively autoregulated, functioning as a repressor that significantly inhibits the expression of the *lrgAB* operon. The *lrgAB* operon is repressed by CdsR in sporulating cells, allowing spore development. However, the CdsR repression of *lrgAB* is released by unknown intracellular signals to activate the expression of LrgAB, which acts as a holin, leading to cell lysis in abnormal cells (Fig. 6). In conclusion, we propose that the CdsR-LrgAB cell death system may be beneficial for the sporulation of *Bacillus* species. This study provides new insights into the relationship between cell survival and death.

## Materials and methods

### Bacterial strains, plasmids, and growth conditions

*Bacillus thuringiensis* HD73 and its derivatives were cultured under 30 °C in Luria-Bertani (LB) medium containing 1% tryptone, 0.5% yeast extract, and 0.5% NaCl or on solid LB medium with 1.5% agar. The growth of bacterial cells was monitored using Schaeffer’s sporulation medium [53] (SSM; 0.8% nutrient broth, 0.012% MgSO_4_, 0.1% KCl, 0.5 mM NaOH, 1 mM Ca(NO_3_)_2_, 0.01 μM MnCl_2_, and 1 μM FeSO_4_). The *Escherichia coli* strains TG1 and ET were, respectively, used for molecular cloning and generation of non-methylated plasmid DNA for subsequent transformation into *B. thuringiensis* strains. Both strains were cultured at 37 °C in LB medium, supplemented with 5 μg/mL of erythromycin and 50 μg/mL of kanamycin for *B. thuringiensis* and with 100 μg/mL of ampicillin for *E. coli*, when necessary. The bacterial strains and plasmids used in this study are listed in Table 1.

**Table 1:** Strains and plasmids used in this study.

Strain or plasmid	Relevant details^a^	Reference or source
*Strains*		
*E. coli* TG1	∆(*lac-proAB*) *supE thi hsd-5* (*F’ traD36 proA*^*+*^ *proB*^*+*^ *lacI*^*q*^ *lacZ*∆M15), general purpose cloning host	Laboratory
*E. coli* ET 12567	*F*^*-*^ *dam-13*::Tn 9 *dcm-6 hsdM hsdR recF143 zjj-202*::Tn10 *galK2 galT22 ara14 pacY1 xyl-5 leuB6 thi-1*, for generation of unmethylated DNA	Laboratory
BL21(DE3)	*F*^*-*^ *dcmopmT hsds* (r_B_^-^ m_B_^-^) galλ(DE3)	[64]
BL21(pET*cdsR*)	BL21(DE3) with pET*cdsR* plasmid	This study
HD73	Wild-type strain containing plasmid pHT73 carrying *cry1Ac* gene	Laboratory
∆*cdsR*	HD73 mutant, *cdsR* gene was deleted by homologous recombination	This study
∆*lrgAB*	HD73 mutant, *lrgAB* operon was deleted by homologous recombination	[44]
C*cdsR*	*cdsR* mutant containing plasmid pHT304-P*cdsR*-*cdsR*	This study
O*lrgAB*	HD73 strain containing plasmid pHT304-P*lrgAB*-*lrgAB*	This study
O*lrgA*	HD73 strain containing plasmid pHT304-P*lrgAB*-*lrgA*	This study
O*lrgB*	HD73 strain containing plasmid pHT304-P*lrgAB*-*lrgB*	This study
HD73(P*cdsR*)	HD73 strain containing plasmid pHT304-P*cdsR*-*lacZ*	This study
∆*cdsR*(P*cdsR*)	*cdsR* mutant containing plasmid pHT304-P*cdsR*-*lacZ*	This study
HD73(P*lrgAB*)	HD73 strain containing plasmid pHT304-P*lrgAB*-*lacZ*	[44]
∆*cdsR*(P*lrgAB*)	*cdsR* mutant containing plasmid pHT304-P*lrgAB*-18Z	This study
HD73(P*cwlD*)	HD73 strain containing plasmid pHT304-P*cwlD*-*lacZ*	This study
∆*cdsR*(P*cwlD*)	*cdsR* mutant containing plasmid pHT304-P*cwlD*-18Z	This study
HD73(P*cwlE*)	HD73 strain containing plasmid pHT304-P*cwlE*-*lacZ*	This study
∆*cdsR*(P*cwlE*)	*cdsR* mutant containing plasmid pHT304-P*cwlE*-18Z	This study
HD73(P*spo0A*)	HD73 strain containing plasmid pHT304-P*spo0A*-*lacZ*	[32]
∆*cdsR*(P*spo0A*)	*cdsR* mutant containing plasmid pHT304-P*spo0A*-*lacZ*	This study
HD73(P*spoIIE*)	HD73 strain containing plasmid pHT304-P*spoIIE*-*lacZ*	[32]
∆*cdsR*(P*spoIIE*)	*cdsR* mutant containing plasmid pHT304-P*spoIIE*-*lacZ*	This study
HD73(P*lrgAB*-*gfp*)	HD73 strain containing plasmid pHT304-P*lrgAB*-*gfp*	This study
∆*lrgAB*(P*lrgAB*-*gfp*)	*lrgAB* mutant containing plasmid pHT304-P*lrgAB*-*gfp*	This study
*Plasmids*		
pHT304-18Z	*E. coli*-*B. thuringiensis* shuttle vector with promoter-less *lacZ* reporter, Amp^R^, Erm^R^	[61]
pHT304	Amp^R^, Erm^R^, *E. coli*-*B. thuringiensis* shuttle vector	[57]
pMAD	Amp^R^, Erm^R^, temperature-sensitive *E. coli*-*B. thuringiensis* shuttle vector	[56]
pET-21b	Expression vector; Amp^R^ 5.4 kb	Laboratory
pET*cdsR*	pET-21b containing *cdsR* gene; Amp^R^	This study
pMAD-∆*cdsR*	pMAD with *cdsR* deletion fragment, Amp^R^, Erm^R^, Kan^R^	This study
pMAD-∆*lrgAB*	pMAD with *lrgAB* deletion fragment, Amp^R^, Erm^R^, Kan^R^	[44]
pHT304-P*cdsR*-*cdsR*	pHT304 carrying P*cdsR* and *cdsR* gene, Amp^R^, Erm^R^	This study
pHT304-P*lrgAB*-*lrgAB*	pHT304 carrying P*lrgAB* and *lrgAB* gene, Amp^R^, Erm^R^	This study
pHT304-P*lrgAB*-*lrgA*	pHT304 carrying P*lrgAB* and *lrgA* gene, Amp^R^, Erm^R^	This study
pHT304-P*lrgAB*-*lrgB*	pHT304 carrying P*lrgAB* and *lrgB* gene, Amp^R^, Erm^R^	This study
pHT304-P*lrgAB*-*gfp*	pHT304 carrying P*lrgAB* and *gfp* gene, Amp^R^, Erm^R^	This study
pHT304P*spo0A*	pHT304-18Z carrying P*spo0A*, Amp^R^, Erm^R^	[32]
pHT304P*spoIIE*	pHT304-18Z carrying P*spoIIE*, Amp^R^, Erm^R^	[44]
pHT304P*cdsR*	pHT304-18Z carrying P*cdsR*, Amp^R^, Erm^R^	This study
pHT304P*cwlD*	pHT304-18Z carrying P*cwlD*, Amp^R^, Erm^R^	This study
pHT304P*cwlE*	pHT304-18Z carrying P*cwlE*, Amp^R^, Erm^R^	This study
pHT304P*lrgAB*	pHT304-18Z carrying P*lrgAB*, Amp^R^, Erm^R^	[44]
p35′*gfp*	pHT315 carrying P*cry35*-*like* and *gfp* gene, Amp^R^, Erm^R^	[58]

^a^Antibiotic resistance cassettes are indicated as follows: Erm^R^, erythromycin resistance; Kan^R^, kanamycin resistance; Amp^R^, ampicillin resistance.

### DNA manipulation and transformation

Plasmid DNA was extracted from *E. coli* cells using the Plasmid Miniprep Kit (Axygen, Beijing, China). Restriction enzymes and T4 DNA ligase (Takara Biotechnology Corporation, Dalian, China) were used according to the manufacturer’s instructions. PCR was performed using either the high-fidelity PrimeStar HS DNA polymerase (Takara Biotechnology Corporation, Beijing, China) or *Taq* DNA polymerase (BioMed, Beijing, China). DNA fragments were purified from 1% agarose gels using the AxyPrep DNA Gel Extraction Kit (Axygen). Standard transformation procedures for *E. coli* [54] and *B. thuringiensis* [55] have been previously described. The primers used in this study are listed in Table 2.

**Table 2:** Oligonucleotide primers used in this study.

Primer name	Sequence (5′–3′)^a^	Restriction site
*cdsR-*AF	GTACCCGGGAGCTCGAATTCTCTTGAGCGAATTGGGC	EcoRI
*cdsR-*AR	TCACCTCAAATGGTTCGCTGATTTTGGCTCATAAAGTTCC	
*cdsR*-KmF	GGAACTTTATGAGCCAAAATCAGCGAACCATTTGAGGTGA	
*cdsR*-KmR	TTCAAATTTTAAGAAAATAAAAATTCCTCGTAGGCGCTCG	
*cdsR-*BF	CGAGCGCCTACGAGGAATTTTTATTTTCTTAAAATTTGAA	
*cdsR-*BR	CGTCGGGCGATATCGGATCCCGTTGTATTCTTCTGGGCAGG	BamHI
C*cdsR*-F	CAGGTCGACTCTAGAGGATCCCGTGATTGTCTGTTAGATACTGC	BamHI
C*cdsR*-R	GTAAAACGACGGCCAGTGAATTCACTCTCTCGCTACCTTTCTGAC	EcoRI
C*lrgAB*-F	CAGGTCGACTCTAGAGGATCCTTACTATCCAATGAATGGTATG	BamHI
C*lrgAB*-R	GTAAAACGACGGCCAGTGAATTCCAAGCGCAAATAGAAACGAAGCACG	EcoRI
C*lrgA*-F	CAGGTCGACTCTAGAGGATCCTGGTTACGCAACCTTTCCGTGCTTG	BamHI
C*lrgA*-R	GTAAAACGACGGCCAGTGAATTCCAAGCGCAAATAGAAACGAAGCACG	EcoRI
CP*lrgAB*-*lrgB*-R	CAAATCGAAAGAGGTGGCCAAACCATTGCATTTATATATTGTTAGGAGG	
CP*lrgAB*-*lrgB*-F	CCTCCTAACAATATATAAATGCAATGGTTTGGCCACCTCTTTCGATTTG	
P*lrgAB*-*gfp*-F	GTCAGAATTCCGCAAATAGAAACGAAGCAC	EcoRI
P*lrgAB*-*gfp*-R	GTTCTTCTCCTTTACTCATTTTGGCCACCTCTTTCGA	
*lrgAB*-*gfp*-F	TCGAAAGAGGTGGCCAAAATGAGTAAAGGAGAAGAAC	
*lrgAB*-*gfp*-R	GTCACTGCAGTTATTTGTATAGTTCATCCATG	PstI
pET*cdsR*-F	CGGGATCCGATGAGCCAAAATCAATTCGAATGTCG	BamHI
pET*cdsR*-R	ACGCGTCGACAGAAAATAATGTTTTTACTACCGCAC	SalI
P*cdsR*-F	AACTGCAGCGTGATTGTCTGTTAGATACTGC	PstI
P*cdsR*-R	CGCGGATCCGCTCATAAAGTTCCCCTC	BamHI
P*cwlD*-F	AACTGCAGGTTCCGGGCTTACAGATGTG	PstI
P*cwlD*-R	CGCGGATCCCATTGTAAATCCTCCTAAG	BamHI
P*cwlE*-F	AACTGCAGGCAATCGCGTCAGCATTGATAGG	PstI
P*cwlE*-R	CGCGGATCCCCATTTTGAAATATCTACAGTGTAACCC	BamHI
RT_*lrgA*_-F	CTTTCCGTGCTTGCGTCCTTTAT	
RT_*lrgA*_-R	ATTGGATTCTTATTCGTCCCATC	
RT_*lrgB*_-F	TTGCTGGCTCTAAGAAGAAACTA	
RT_*lrgB*_-R	AATCGCATACGGAATCGGAACAT	
RT_*16S*_-F	TCGCATTAGCTAGTTGGTGAG	
RT_*16S*_-R	TCTTCCCTAACAACAGAGTTT	

^a^Restriction enzyme sites are underlined.

### Construction of the *cdsR* deletion mutant

The primers used for gene deletion were designed based on the HD73 genome sequence. A 1032-bp fragment preceding the start codon of *cdsR* (*cdsR* fragment A) was amplified by PCR, utilizing HD73 genomic DNA as the template and *cdsR*-AF and *cdsR*-AR as the forward and reverse primers, respectively. Furthermore, the primers *cdsR*-BF and *cdsR*-BR were used to amplify a 1015-bp fragment downstream of the 96th codon of *cdsR* (*cdsR* fragment B), whereas the primers *cdsR*-KmF and *cdsR*-KmR were utilized to amplify a 1473-bp kanamycin resistance gene cassette (kana) derived from pMAD-Δ*lrgAB* [44] plasmid. The *cdsR* A, kana, and *cdsR* B fragments were ligated through overlapping PCR using the primers *cdsR*-AF and *cdsR*-BR. The 3568-bp overlapping fragment was inserted between the BamHI and EcoRI restriction sites of the temperature-sensitive and erythromycin-resistant plasmid pMAD [56], resulting in the generation of the plasmid pMAD-Δ*cdsR*, which was then electroporated into HD73 using the Gene Pulser II apparatus (Bio-Rad, Hercules, CA, USA). Subsequently, transformants were selected for erythromycin resistance at 30 °C. The positive transformants were transferred to 37 °C and screened for the colonies lacking erythromycin resistance but resistant to kanamycin to obtain the mutant Δ*cdsR*, which was characterized by PCR.

### Construction of multiple strains

The *cdsR* gene fragment and *cdsR* promoters were amplified with the C*cdsR*-F/C*cdsR*-R primers using HD73 genomic DNA as a template. The resulting 969-bp PCR product was inserted into plasmid pHT304 [57] using EcoRI and BamHI to obtain pHT304-P*cdsR*-*cdsR*. This recombinant construct was replicated in *E. coli* and subsequently introduced into the Δ*cdsR* mutant, resulting in a genetically complementary strain C*cdsR*.

Two fragments were amplified using HD73 genomic DNA as a template: one was a 1676-bp fragment containing the *lrgAB* operon and its promoter region, amplified with the C*lrgAB*-F/C*lrgAB*-R primers, and the other was a 948-bp fragment containing the *lrgA* gene and its promoter region, amplified with the C*lrgA*-F/C*lrgA*-R primers. The *lrgAB* operon’s promoter region (513-bp) was amplified with the C*lrgAB*-F/CP*lrgAB*-*lrgB*-R primers and the *lrgB* gene fragment (731-bp) was amplified with the CP*lrgAB*-*lrgB*-F/C*lrgAB*-R primers, using HD73 genomic DNA as a template. The above two fragments were combined to create a 1244-bp fragment through overlapping PCR using the C*lrgAB*-F/C*lrgAB*-R primers. The fragments of sizes 1676, 948, and 1244 bp were individually ligated into plasmid pHT304, using the EcoRI and BamHI sites, to obtain the recombinant plasmids pHT304-P*lrgAB*-*lrgAB*, pHT304-P*lrgAB*-*lrgA*, and pHT304-P*lrgAB*-*lrgB*, respectively. These recombinant plasmids were then introduced into the HD73 strain to obtain the O*lrgAB*, O*lrgA*, and O*lrgB* strains for overexpression, respectively.

The strains in which the promoter of the *lrgAB* operon directs *gfp* gene expression were constructed as follows: HD73 genomic DNA served as a template to amplify the *lrgAB* promoter fragment (P*lrgAB*, 528-bp) with the P*lrgAB*-*gfp*-F/P*lrgAB*-*gfp*-R primers. The *gfp* gene (735-bp) was amplified using the plasmid p35′*gfp* [58] as a template with the *lrgAB*-*gfp*-F/*lrgAB*-*gfp*-R primers. The P*lrgAB* and *gfp* fragments were used as templates to jointly amplify the P*lrgAB*-*gfp* gene fusion fragment (1226-bp) using the P*lrgAB*-*gfp*-F/*lrgAB*-*gfp*-R primers. The resulting PCR product was inserted into the plasmid pHT304 through the EcoRI and PstI sites, leading to the generation of the plasmid pHT304-P*lrgAB*-*gfp*, which, in turn, was introduced into both the HD73 and Δ*lrgAB* mutant strains [44].

### Autolysis observation and autolytic activity

To determine the bacterial culture density, a 5 mL sample was cultured in 50 mL SSM medium for 21 h and then transferred to a glass test tube. The strains HD73, Δ*cdsR*, and C*cdsR* were measured by the OD_600_ value, using three independent biological replicates.

The 5 mL samples collected from the 50 mL SSM at T_6_ (T_0_ indicates the end of the exponential growth phase, and T_n_ indicates the number of hours before (-) or after T_0_) were centrifuged at 12,000 × *g* for 10 min at 4 °C. The resulting pellets were washed thrice with 50 mL of sterile water and resuspended in 30 mL of 0.05 M Tris-HCl (pH 7.2) containing 0.05% (vol/vol) Triton X-100 (Sigma Chemical Company, St. Louis, MO, USA) [59]. Subsequently, the bacteria cells were incubated in a shaking incubator at 220 rpm and 30 °C. The OD_580_ values of the samples were measured every 1 h from A_1_ to A_8_ (with A_0_ denoting the initial value of cells at T_6_ and A_n_ indicating the number of hours after time A_0_).

### Microscope observation

*Bacillus thuringiensis* cells were cultured in SSM, and samples were collected at specified time points for analysis. Transmission electron microscopy (TEM) was used to observe the bacterial cell and spore morphology [60]. Optical microscopy was performed to characterize cell types using an Olympus BX63 microscope. Confocal microscopy (Carl Zeiss 880) was performed to examine polar septum formation, spore engulfment processes, and cell viability. Various fluorescent dyes, including FM4-64 (100 μM), MitoTracker Green (MTG) (100 nM), and SYTOX^TM^ Green (5 nM), were used for these observations.

### Determination of cell number

Samples of *B. thuringiensis* cells, including HD73 and Δ*lrgAB* mutant cultured for T_24_, were collected for dilution and plating. The total number of cells was determined by counting the total number of colony-forming units (CFUs) on LB plates.

The number of total cells and dead cells in the microscopic images were counted. Optical or laser confocal microscopy images were used to quantify at least 500 cells of each strain type. The cell death rate was defined as the ratio of the number of dead cells (stained with SYTOX^TM^ Green) to the total number of cells. The cell death rate was determined from at least three independent cultures. Moreover, the abnormal and GFP-expressing cell rates were statistically calculated in a similar manner.

### Transcriptome sequencing and analysis

The HD73 and Δ*cdsR* mutant strains were cultured in SSM at 30 °C and harvested at T_0_. Total RNA was extracted using the RNAprep Pure Bacteria Kit (Aidlab, Beijing, China). RNA sequencing (RNA-Seq) was carried out by SinoBiocore (Beijing, China). Ribosomal RNA was removed using the Ribo Zero™ rRNA Removal Kit (Epicentre, Madison, WI, USA), and rRNA-free residue was removed by ethanol precipitation. Subsequently, the ribosome-depleted mRNA was fragmented and reverse transcribed. The first-strand cDNA was synthesized using random hexamer primers and M-MuLV Reverse Transcriptase, and the second-strand cDNA was synthesized using DNA Polymerase I and RNase H. For the dNTPs in the reaction buffer, the dTTPs were replaced with dUTPs. After adenylation of 3′ ends of DNA fragments, the NEBNext Adaptors (New England BioLabs, Ipswich, MA, USA) with a hairpin loop structure were ligated to prepare for hybridization. To select cDNA fragments of preferentially 250–300 bp in length, the library fragments were purified using the AMPure XP system (Beckman Coulter, Beverly, CA, USA). Subsequently, 3 µL of the USER Enzyme (New England BioLabs) was used with size-selected, adaptor-ligated cDNA at 37 °C for 15 min followed by 5 min at 95 °C before PCR. The PCR was performed using the Phusion High-Fidelity DNA Polymerase, universal PCR primers, and index (X) primers. The resulting PCR products were purified using the AMPure XP system, and the quality of the libraries was assessed using an Agilent Bioanalyzer 2100 system (Agilent Technologies, Palo Alto, CA, USA). The clustering of index-coded samples was carried out on a cBot Cluster Generation System (Illumina, USA). Library preparations were sequenced on an Illumina HiSeq X Ten platform with a 150-bp paired-end module. The sequence reads were mapped to the HD73 reference genome (NC_020238.1).

The relative transcript abundance was calculated as fragments per kilobase of exon sequence per million mapped sequence reads (FPKM). Genes were considered differentially expressed only if they met the criteria of having an adjusted *P*-value ≤ 0.05 and absolute value of log_2_ fold-change ≥1. The differentially expressed genes were subjected to the Kyoto Encyclopedia of Genes and Genomes (KEGG) pathway enrichment analysis using an online gene function analysis tool. All RNA-Seq data were uploaded to the Gene Expression Omnibus database of the National Center for Biotechnology Information (NCBI; www.ncbi.nlm.nih.gov) (accession no. GSE216307).

### β-galactosidase activity assays

The promoter sequence of *cdsR* (614-bp upstream of the *cdsR* translational start codon) was amplified from HD73 genomic DNA using the P*cdsR*-F and P*cdsR*-R primers. The resulting P*cdsR* fragments were then digested with PstI and BamHI and ligated into the linearized pHT304-18Z plasmid, which harbors a promoter-less *lacZ* gene [61], to obtain the recombinant plasmid pHT304P*cdsR*. Subsequently, the recombinant plasmid was introduced into both HD73 and Δ*cdsR* strains, leading to the generation of the strains HD(P*cdsR*) and Δ*cdsR*(P*cdsR*), respectively, which were validated by erythromycin resistance and PCR identification. To assess the transcription of *spo0A*, *spoIIE*, *lrgAB*, *cwlD,* and *cwlE* in HD73 and Δ*cdsR*, a similar methodology was employed to construct the desired strains. The recombinant plasmids pHT304P*spo0A* [32], pHT304P*spoIIE* [44], and pHT304P*lrgAB* [44] were introduced into both HD73 and Δ*cdsR* strains. The P*cwlD* promoter (592-bp) and P*cwlE* promoter (418-bp) were amplified from HD73 genomic DNA using the primers P*cwlD*-F/P*cwlD*-R and P*cwlE*-F/P*cwlE*-R, respectively. Following amplification, these promoter fragments were digested with PstI and BamHI and subsequently ligated into the linearized pHT304-18Z plasmid to obtain recombinant plasmids pHT304P*cwlD* and pHT304P*cwlE*, which were then introduced into HD73 and Δ*cdsR*, respectively. The β-galactosidase activities were measured as previously described [62] and expressed as Miller units. Samples were collected at 1-h intervals from T_−2_ to T_6_. For each sample, 2 mL of culture was centrifuged (12,000 × *g*, 1 min), and the resulting pellets were stored at −20 °C until testing. The reported values represent the mean and standard error of at least three independent assays.

### Expression and purification of CdsR

The expression plasmid pET*cdsR*, containing the *cdsR* gene from HD73, was constructed by amplifying with the primers pET*cdsR*-F and pET*cdsR*-R, followed by cloning into BamHI/SalI-digested pET21b. The resulting pET*cdsR* plasmid was transferred into *E. coli* BL21 (DE3), and the transformants were grown to the exponential phase in LB medium supplemented with ampicillin at 37 °C. Subsequently, the expression of CdsR-His protein was induced by adding 1 mM isopropyl-β-D-thiogalactopyranoside (final concentration) to the culture for 12 h at 18 °C. The resulting CdsR-His protein was purified as previously described [63].

### Electrophoresis mobility shift assays

The DNA fragments were PCR-amplified from HD73 genomic DNA using specific primers labeled with a 5′-end fluorescein amidite (FAM) modification and confirmed by DNA sequencing. Electrophoretic mobility shift assays (EMSA) [31] were performed as previously described to analyze the binding between the purified CdsR protein and P*lrgAB*, P*cdsR*, and P*cwlD* DNA fragments.

### Real-time quantitative PCR assay

Cells expressing O*lrgAB*, O*lrgA*, and O*lrgB* were harvested to analyze the expression levels of *lrgA* and *lrgB* at T_6_. Total RNA was extracted from the collected cells using the RNAprep Pure Kit (Aidlab, Beijing, China). Subsequently, cDNA was synthesized by reverse transcription using the HiScript II Q RT SuperMix (Vazyme, Nanjing, China). The cDNA concentration was diluted to 200 ng/μL. The transcriptional levels of the target genes were determined by real-time quantitative PCR (RT-qPCR) using the ChamQ Universal SYBR qPCR master mix (Vazyme). The 16S rRNA gene was used as an internal control. The primers used for RT-qPCR analysis are listed in Table 2.

### Bioinformatics analysis

The CdsR homologous protein sequences obtained from NCBI were used for the phylogenetic tree analysis. Multiple sequence alignments were performed and visualized using the software programs ClustalW and GeneDoc 3.2, respectively, and a phylogenetic tree was constructed using the neighbor-joining method in the MEGA 7.0 software. Furthermore, the homologous protein sequences of LrgAB were retrieved from the NCBI to analyze interspecies distributions and perform similarity comparisons.

### Statistical analysis

Data are means ± sem. Significant differences were determined with a Student’s T-test with *P < 0.05; **P < 0.01; ***P < 0.001; and ****P < 0.0001 (*n* ≥ 3). Statistical analysis was performed using Excel and GraphPad Prism 9 software. A *P*-value of less than 0.05 was considered significant for all tests.

### Supplementary information

supplementary figure and table

## Data Availability

We have uploaded the DNA microarray and RNA-Seq data, which will be available in the NCBI Gene Expression Omnibus database with the accession no. GSE48410 and GSE216307. All data generated or analyzed during this study are included in this published article.
