# Supplementary material for: Cell death dependent on holins LrgAB repressed by a novel ArsR family regulator CdsR
Source: Cell Death Discov. 2024 Apr 11;10:173. doi: 10.1038/s41420-024-01942-3 (PMC11009283; doi:10.1038/s41420-024-01942-3)
Supplement: Supplementary file 1 — supplementary figure and table [file 41420_2024_1942_MOESM1_ESM.pdf]

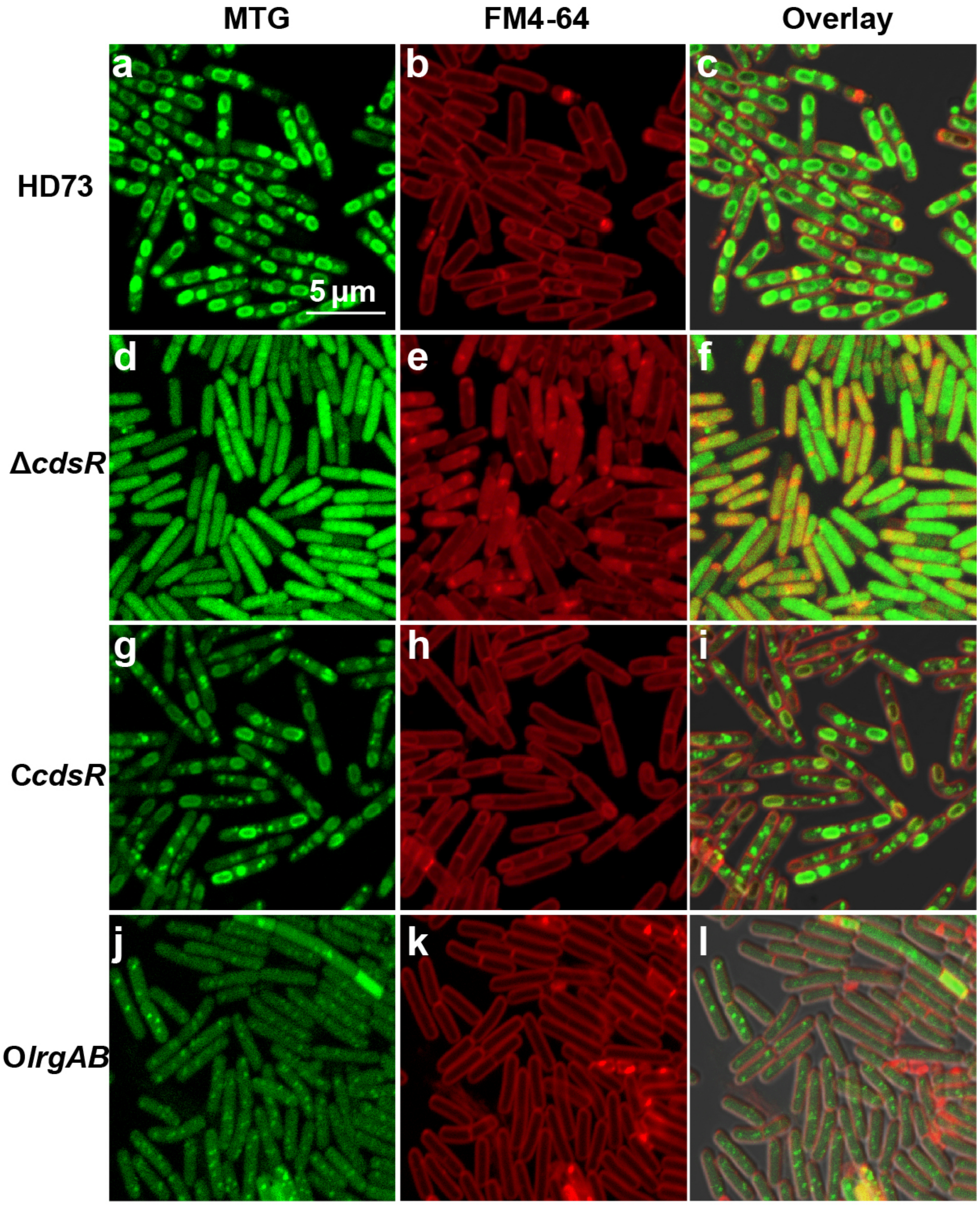

Figure S1. Laser scanning confocal microscopy of spore engulfment

The HD73, Δ*cdsR*, C*cdsR*, and *OlrgAB* strains were cultured to T_7 in SSM. Red outline represent the membranes stained with FM4-64, and MitoTracker green (MTG) dyes were used to stain the forespores.

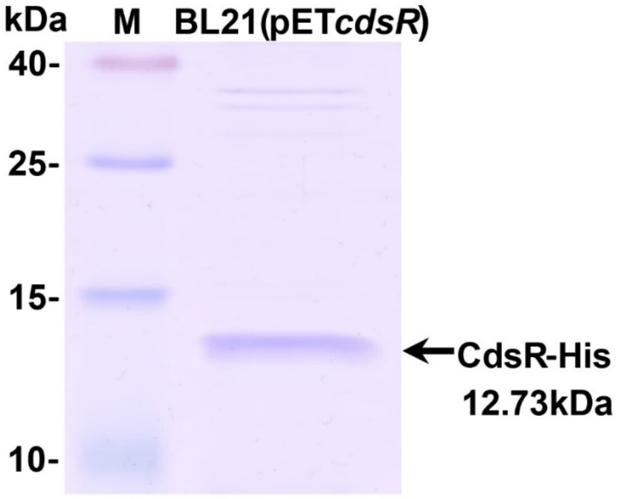

Figure S2. Purification of the CdsR-His recombinant protein

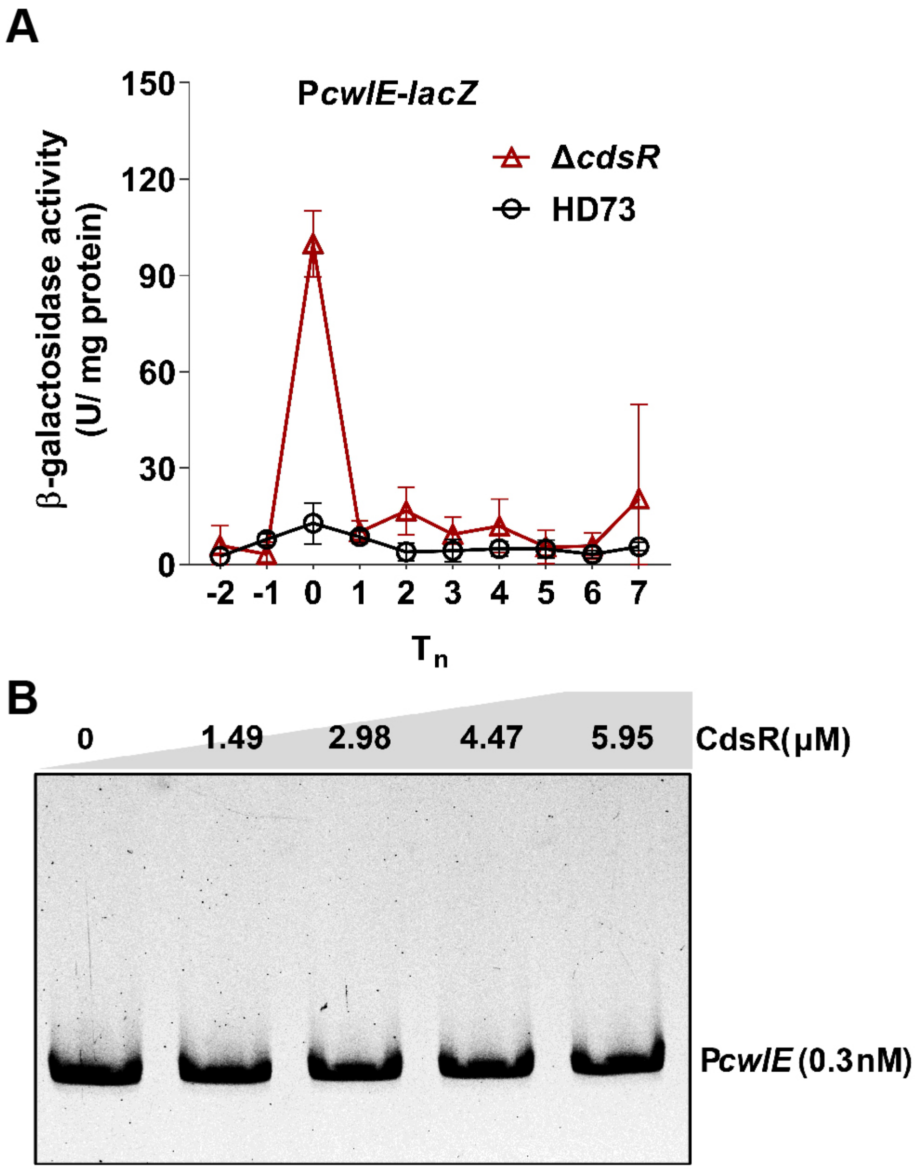

Figure S3. Analysis of the regulatory effect of CdsR on *cwlE* expression

(A) Transcription analysis of the *cwlE* promoter in HD73 and Δ*cdsR*. The β-galactosidase activities of three clones were determined at the specified time points after cultivating *Bacillus thuringiensis* cells in SSM at 30°C. Each value represents the mean and standard error of at least three independent replicates. (B) Interaction between CdsR and *cwlE* promoter (P*cwlE*) was assessed by electrophoretic mobility shifty assay (EMSA). The increasing amounts of the purified CdsR were incubated with the *cwlE* promoter regions.

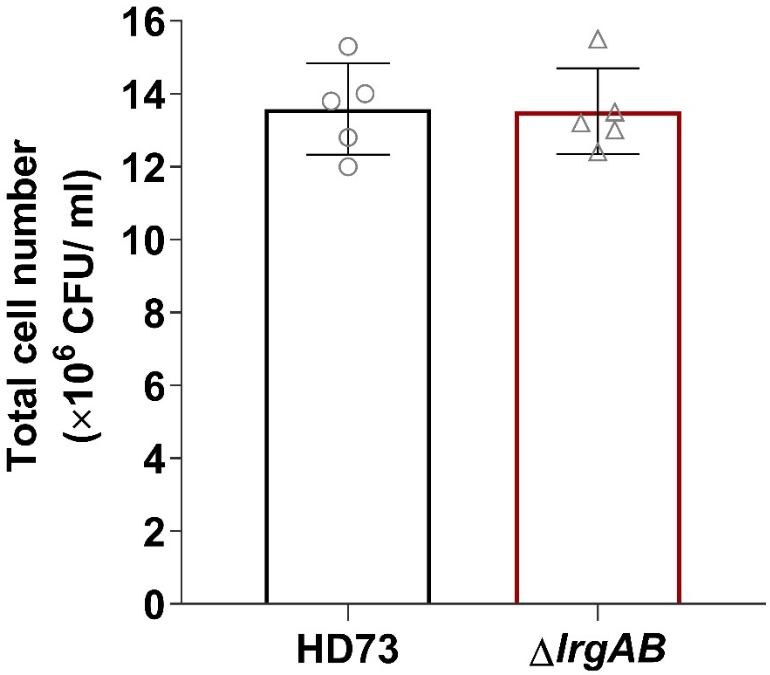

Figure S4. Cell counts of HD73 and Δ*lrgAB* cultures were determined on LB plates.
Sample cells were cultured in SSM medium at T_{24} and the total number of cells was determined by serial dilution and plating. Data were obtained from five independent experiments.

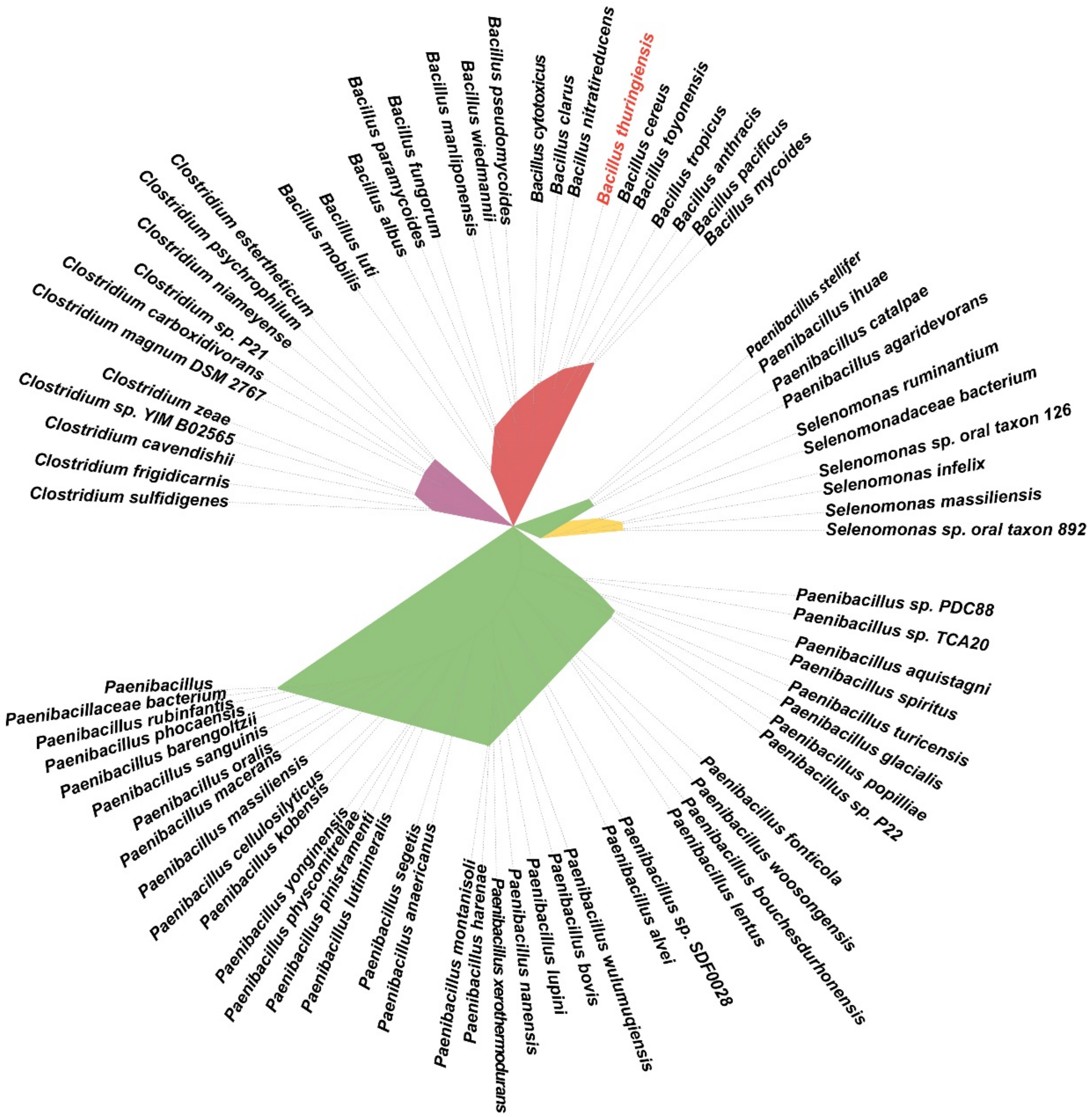

Figure S5. Diversity analysis of CdsR homologs

The phylogenetic tree includes 75 CdsR homologs with E-values below 1E-50. A purple background was used as a reference to identify the CdsR homologs in *B. thuringiensis* HD73 using BLASTP. The optimal tree with a sum of branch lengths of 8.67394886 is shown. The analysis included 75 amino acid sequences, with gaps or missing data positions excluded. Evolutionary analyses were conducted using MEGA version 7.0. A list of the bacterial species is provided in Supplementary Table 4.

SUPPLEMENTARY TABLE 1 Microarray data for the top 30 unknown function regulators with high expression on the stationary phase

No.	Gene ID	Relative expression level	annotation
**1**	HD73_RS24960	15.710121	DeoR-family transcriptional regulator
**2**	HD73_RS22725	14.42169033	ArsR-family transcriptional regulator
**3**	HD73_RS06550	14.098301	LacI-family transcriptional regulator
**4**	HD73_RS22445	13.29530767	DtxR-family transcriptional regulator
**5**	HD73_RS08015	13.009614	PadR-family transcriptional regulator
**6**	HD73_RS20220	12.48677767	Xre-family transcriptional regulator
**7**	HD73_RS11500	12.320244	Xre-family transcriptional regulator
**8**	HD73_RS03760	11.96522833	ArsR-family transcriptional regulator
**9**	HD73_RS18030	11.91154467	MerR family transcriptional regulator
**10**	HD73_RS19255	11.88471133	transcriptional regulator, unclassified
**11**	HD73_RS26800	11.61916167	TetR-family transcriptional regulator
**12**	HD73_RS24985	11.55706467	ArsR-family transcriptional regulator
**13**	HD73_RS21920	11.54903933	LacI-family transcriptional regulator
**14**	HD73_RS22020	11.53921967	DNA-binding protein, unclassified
**15**	HD73_RS17170	11.4117775	MerR-family transcriptional regulator
**16**	HD73_RS10040	11.374027	MerR-family transcriptional regulator
**17**	HD73_RS15065	11.26833233	IclR-family transcriptional regulator
**18**	HD73_RS20105	10.82612167	GntR-family transcriptional regulator
**19**	HD73_RS10365	10.8198565	Mor-family transcriptional regulator
**20**	HD73_RS27770	10.71242233	Xre-family transcriptional regulator
**21**	HD73_RS16165	10.678558	Rrf2-family transcriptional regulator
**22**	HD73_RS08130	10.67405867	MarR-family transcriptional regulator
**23**	HD73_RS23765	10.65675833	MarR-family transcriptional regulator
**24**	HD73_RS22910	10.62974133	transcriptional regulator, unclassified

**25**	HD73_RS04085	10.60013817	Xre-family transcriptional regulator
**26**	HD73_RS24395	10.581313	GntR-family transcriptional regulator
**27**	HD73_RS22490	10.53493467	TetR-family transcriptional regulator
**28**	HD73_RS06715	10.50320867	AraC-family transcriptional regulator
**29**	HD73_RS08390	10.44867633	MarR-family transcriptional regulator
**30**	HD73_RS27785	10.44293567	DNA-binding protein, unclassified

SUPPLEMENTARY TABLE 2 Functional classification of down- and upregulated genes expressed at T_0 in Δ*cdsR*.

Genes ID	Annotations	Log_2(foldchange)
**Amino acid metabolism**		
**Upregulated genes**		
HD73_RS10405	acetolactate synthase large subunit	3
HD73_RS10400	branched-chain-amino-acid transaminase	2.92
HD73_RS10410	ACT domain-containing protein	2.89
HD73_RS25815	L-lactate dehydrogenase	2.67
HD73_RS10415	ketol-acid reductoisomerase	2.66
HD73_RS10420	dihydroxy-acid dehydratase	2.66
HD73_RS30450	AimR family lysis-lysogeny pheromone receptor	2.65
HD73_RS23295	homocysteine desulfhydrase	2.53
HD73_RS10915	homoserine dehydrogenase	2.39
HD73_RS25410	S-ribosylhomocysteine lyase LuxS	2.31
HD73_RS04505	amino acid permease	2.29
HD73_RS08470	3-isopropylmalate dehydrogenase	2.28
HD73_RS10425	threonine ammonia-lyase IlvA	2.28
HD73_RS08460	ketol-acid reductoisomerase	2.24
HD73_RS08455	acetolactate synthase small subunit	2.06
HD73_RS22165	amino acid ABC transporter permease	2.05
HD73_RS10920	threonine synthase	2.04
HD73_RS24345	NADP-dependent isocitrate dehydrogenase	2.02
HD73_RS22655	L-cystine transporter	2.01
HD73_RS08475	3-isopropylmalate dehydratase large subunit	1.98
HD73_RS08450	acetolactate synthase large subunit	1.96
HD73_RS08465	2-isopropylmalate synthase	1.94
HD73_RS03880	alanine:cation symporter family protein	1.93
HD73_RS10925	homoserine kinase	1.92
HD73_RS03915	OsmC family protein	1.84
HD73_RS22160	amino acid ABC transporter ATP-binding protein	1.81
HD73_RS08480	3-isopropylmalate dehydratase small subunit	1.77
HD73_RS08915	NAD-specific glutamate dehydrogenase	1.73
HD73_RS07625	Aminodeoxychorismate /anthranilate synthase component II	1.71
HD73_RS06880	ornithine aminotransferase	1.69
HD73_RS08445	branched-chain-amino-acid transaminase	1.66
HD73_RS22170	transporter substrate-binding domain-containing protein	1.6
HD73_RS16770	serine/threonine protein kinase	1.56
HD73_RS23305	5'-methylthioadenosine/S-adenosylhomocysteine nucleosidase	1.56

HD73_RS24340	malate dehydrogenase	1.56
HD73_RS00385	Cysteine synthase A	1.54
HD73_RS23300	O-acetylserine dependent cystathionine beta-synthase	1.5
HD73_RS10390	peptide-methionine (S)-S-oxide reductase MsrA	1.49
HD73_RS21435	acetyl-CoA C-acetyltransferase	1.45
HD73_RS04445	amino acid permease	1.44
HD73_RS15245	bifunctional 3-deoxy-7-phosphoheptulonate synthase/chorismate mutase	1.44
HD73_RS03910	amino acid permease	1.43
HD73_RS26785	L-lactate dehydrogenase	1.43
HD73_RS03840	amino acid permease	1.4
HD73_RS08505	imidazole glycerol phosphate synthase subunit HisH	1.39
HD73_RS08110	cyclic di-AMP binding protein CbpA	1.38
HD73_RS08105	class II aldolase/adducin family protein	1.37
HD73_RS08485	ATP phosphoribosyltransferase regulatory subunit	1.36
HD73_RS07630	anthranilate phosphoribosyltransferase	1.34
HD73_RS09180	3-methyl-2-oxobutanoate hydroxymethyltransferase	1.33
HD73_RS10930	DUF6366 family protein	1.31
HD73_RS05525	proline racemase family protein	1.3
HD73_RS09190	aspartate 1-decarboxylase	1.3
HD73_RS12905	lysine 2,3-aminomutase	1.29
HD73_RS07640	phosphoribosylanthranilate isomerase	1.28
HD73_RS08495	histidinol dehydrogenase	1.27
HD73_RS24525	argininosuccinate lyase	1.27
HD73_RS26845	MTH1187 family thiamine-binding protein	1.27
HD73_RS08515	imidazoleglycerol phosphate synthase cyclase subunit	1.21
HD73_RS22420	type II 3-dehydroquinate dehydratase	1.21
HD73_RS09185	pantoate--beta-alanine ligase	1.19
HD73_RS23310	class I SAM-dependent methyltransferase	1.18
HD73_RS07645	tryptophan synthase subunit beta	1.15
HD73_RS08490	ATP phosphoribosyltransferase	1.13
HD73_RS15815	proline racemase family protein	1.13
HD73_RS08510	1-(5-phosphoribosyl)-5-[(5-phosphoribosylamino) methylideneamino] imidazole-4- carboxamide isomerase	1.12
HD73_RS15810	proline racemase family protein	1.12
HD73_RS20305	aspartate kinase	1.12
HD73_RS07635	indole-3-glycerol phosphate synthase TrpC	1.11
HD73_RS03800	glycine C-acetyltransferase	1.1
HD73_RS09030	2-heptaprenyl-1,4-naphthoquinone methyltransferase	1.09
HD73_RS04515	amidohydrolase	1.08
HD73_RS24530	argininosuccinate synthase	1.08

HD73_RS08500	imidazoleglycerol-phosphate dehydratase HisB	1.07
HD73_RS02045	L-glutamate gamma-semialdehyde dehydrogenase	1.03
HD73_RS09050	3-dehydroquinate synthase	1.03
HD73_RS21335	5-methyltetrahydropteroyltriglutamate-homocysteine S-methyltransferase	1.02
HD73_RS20310	aspartate-semialdehyde dehydrogenase	1.01
	**Amino acid metabolism**	
	**Downregulated genes**	
HD73_RS26585	cysteine protease StiP family protein	-2.35
HD73_RS23225	aromatic amino acid hydroxylase	-2.2
HD73_RS10260	aspartate kinase	-1.92
HD73_RS14055	class I SAM-dependent methyltransferase	-1.58
HD73_RS28310	CpsD/CapB family tyrosine-protein kinase	-1.29
HD73_RS12600	serine hydrolase	-1.22
HD73_RS17515	L-serine ammonia-lyase, iron-sulfur-dependent, subunit alpha	-1.15
HD73_RS02690	arginine deiminase	-1.07
HD73_RS15230	cysteine hydrolase	-1.06
HD73_RS17520	serine dehydratase	-1.03
	**Autolysis**	
	**Upregulated genes**	
HD73_RS29145	antiholin-like protein LrgB	3.28
HD73_RS29150	antiholin-like murein hydrolase modulator LrgA	2.75
HD73_RS20125	LysM peptidoglycan	2.33
HD73_RS04330	GH25 family lysozyme	2.08
	**Autolysis**	
	**Downregulated genes**	
HD73_RS26205	N-acetylmuramoyl-L-alanine amidase	-6.35
HD73_RS12685	N-acetylmuramoyl-L-alanine amidase	-1.09
	**Carbohydrate metabolism**	
	**Upregulated genes**	
HD73_RS24350	citrate synthase	2.21
HD73_RS03075	N-acetylglucosamine-specific PTS transporter subunit IIBC	1.99
HD73_RS10575	NupC/NupG family nucleoside CNT transporter	1.99
HD73_RS10650	VOC family protein	1.97
HD73_RS10570	deoxyribose-phosphate aldolase	1.81
HD73_RS21660	N-acetylglucosamine-6-phosphate deacetylase	1.73
HD73_RS07970	ykgG family protein	1.72
HD73_RS02745	endonuclease/exonuclease/phosphatase	1.66
HD73_RS07735	2-oxoglutarate dehydrogenase complex	1.54

	dihydrolipoyllysine-residue succinyltransferase	
HD73_RS11085	NUDIX hydrolase	1.54
HD73_RS02740	PTS transporter subunit IIBC	1.53
HD73_RS10580	pyrimidine-nucleoside phosphorylase	1.52
HD73_RS28110	glycosyltransferase family 2 protein	1.5
HD73_RS03565	malate dehydrogenase	1.49
HD73_RS21655	glucosamine-6-phosphate deaminase	1.45
HD73_RS10585	cytidine deaminase	1.39
HD73_RS22240	phosphate butyryltransferase	1.35
HD73_RS05200	PTS transporter subunit EIIC	1.32
HD73_RS07965	iron-sulfur cluster-binding protein	1.27
HD73_RS08010	polyhydroxyalkanoic acid inclusion protein PhaP	1.24
HD73_RS15180	malate:quinone oxidoreductase	1.19
HD73_RS26765	arsenate reductase family protein	1.16
HD73_RS26760	glycine cleavage system protein GcvH	1.15
HD73_RS21105	inositol monophosphatase family protein	1.1
HD73_RS07740	2-oxoglutarate dehydrogenase E1 component	1.09
HD73_RS06775	isocitrate lyase	1.09
HD73_RS08005	MaoC family dehydratase	1.05
	**Carbohydrate metabolism**	
	**Downregulated genes**	
HD73_RS17335	acetyl-CoA carboxylase biotin carboxyl carrier protein subunit	-4.17
HD73_RS16155	dihydrolipoyl dehydrogenase	-4
HD73_RS17340	acetyl-CoA carboxylase biotin carboxylase subunit	-3.96
HD73_RS16145	acetoin:2,6-dichlorophenolindophenol	-3.53
HD73_RS17315	AMP-binding protein	-3.51
HD73_RS17345	acyl-CoA dehydrogenase	-3.29
HD73_RS17330	hydroxymethylglutaryl-CoA lyase	-3.18
HD73_RS17320	acyl-CoA carboxylase subunit beta	-3.15
HD73_RS17325	enoyl-CoA hydratase	-3.02
HD73_RS31785	DUF4085 domain-containing protein	-3.01
HD73_RS16150	2-oxo acid dehydrogenase subunit E2	-2.86
HD73_RS09935	histidine phosphatase family protein	-1.66
HD73_RS21475	3-hydroxybutyrate dehydrogenase	-1.49
HD73_RS02120	4-aminobutyrate--2-oxoglutarate transaminase	-1.44
HD73_RS19050	aldehyde dehydrogenase DhaS	-1.12
HD73_RS12950	phosphatase PAP2 family protein	-1.11
HD73_RS16035	ribose 5-phosphate isomerase A	-1.07
HD73_RS13105	CoA-acylating methylmalonate-semialdehyde dehydrogenase	-1

Cell motility		
**Upregulated genes**		
HD73_RS04395	methyl-accepting chemotaxis protein	2.8
HD73_RS09650	YaaR family protein	2.74
HD73_RS09825	Flagellar basal-body rod protein flgG	2.41
HD73_RS26970	methyl-accepting chemotaxis protein	2.37
HD73_RS09620	chemotaxis protein CheA	2.34
HD73_RS09705	flagellar motor switch protein FliG	2.33
HD73_RS09660	Flagellar hook-associated protein FlgK	2.32
HD73_RS26975	methyl-accepting chemotaxis protein	2.31
HD73_RS09805	flagellar biosynthetic protein FliR	2.3
HD73_RS09625	flagellar motor switch protein	2.29
HD73_RS09680	hypothetical protein	2.27
HD73_RS23930	flagellar motor stator protein MotA	2.24
HD73_RS09635	hypothetical protein	2.23
HD73_RS09615	response regulator	2.15
HD73_RS23925	flagellar motor protein MotB	2.14
HD73_RS09750	chemotaxis protein	2.13
HD73_RS09675	flagellar protein FliS	2.13
HD73_RS09730	flagellar hook assembly protein FlgD	2.07
HD73_RS09630	hypothetical protein	2.07
HD73_RS09790	hypothetical protein	2.01
HD73_RS09700	flagellar M-ring protein FliF	1.99
HD73_RS09655	hypothetical protein	1.98
HD73_RS09640	protein-glutamate O-methyltransferase CheR	1.98
HD73_RS09785	flagellar motor switch protein FliN	1.97
HD73_RS09795	flagellar type III secretion system pore protein FliP	1.97
HD73_RS09665	Flagellar hook-associated protein 3	1.96
HD73_RS09740	DUF3964 family protein	1.95
HD73_RS09690	flagellar basal body rod protein FlgC	1.93
HD73_RS09605	flagellar motor protein MotP	1.87
HD73_RS09670	Flagellar hook-associated protein 2	1.86
HD73_RS09610	OmpA family protein	1.83
HD73_RS09725	flagellar hook-length control protein FliK	1.82
HD73_RS09735	flagellar hook protein FlgE	1.81
HD73_RS09715	flagellar protein export ATPase FliI	1.74
HD73_RS09685	flagellar basal body rod protein FlgB	1.67
HD73_RS09645	hypothetical protein	1.63
HD73_RS09770	transglycosylase SLT domain-containing protein	1.58
HD73_RS09780	flagellar motor switch protein FliM	1.57
HD73_RS09720	hypothetical protein	1.55

HD73_RS09745	hypothetical protein	1.5
HD73_RS09800	flagellar biosynthetic protein FliQ	1.45
HD73_RS18820	methyl-accepting chemotaxis protein	1.45
HD73_RS09775	flagellar motor switch protein FliN	1.33
HD73_RS09820	flagellar biosynthesis protein FlhF	1.32
HD73_RS02680	methyl-accepting chemotaxis protein	1.16
HD73_RS09710	flagellar assembly protein FliH	1.07
	**Energy metabolism**	
	**Upregulated genes**	
HD73_RS12090	DUF58 domain-containing protein	2.36
HD73_RS03655	heavy metal translocating P-type ATPase	2.07
HD73_RS12620	FMN-dependent NADH-azoreductase	2.04
HD73_RS18020	ATP synthase subunit I	2.02
HD73_RS03220	glutamate synthase-related protein	2.01
HD73_RS12155	nitrate transporter NarK	1.94
HD73_RS12095	MoxR family ATPase	1.94
HD73_RS12085	DUF3488 and DUF4129 domain-containing transglutaminase family protein	1.94
HD73_RS27065	pyrimidine nucleoside transporter NupC	1.77
HD73_RS24595	NAD kinase	1.75
HD73_RS26480	NAD(P)/FAD-dependent oxidoreductase	1.68
HD73_RS11220	GNAT family N-acetyltransferase	1.65
HD73_RS15300	LTA synthase family protein	1.63
HD73_RS12120	respiratory nitrate reductase subunit gamma	1.44
HD73_RS12180	nitrite reductase small subunit NirD	1.42
HD73_RS04500	cytochrome aa3 quinol oxidase subunit II	1.4
HD73_RS12115	nitrate reductase molybdenum cofactor assembly chaperone	1.39
HD73_RS15120	heme-degrading monooxygenase HmoA	1.38
HD73_RS09020	GTP cyclohydrolase I FolE	1.36
HD73_RS07270	Cof-type HAD-IIB family hydrolase	1.35
HD73_RS11395	cyclic di-AMP binding protein CbpA	1.34
HD73_RS15785	NAD(P)/FAD-dependent oxidoreductase	1.34
HD73_RS07310	beta-ketoacyl-ACP synthase III	1.32
HD73_RS10445	GNAT family N-acetyltransferase	1.3
HD73_RS13810	non-ribosomal peptide synthetase	1.23
HD73_RS11205	LLM class flavin-dependent oxidoreductase	1.21
HD73_RS05330	NADP-dependent glyceraldehyde-3-phosphate dehydrogenase	1.21
HD73_RS21830	purine-nucleoside phosphorylase	1.18
HD73_RS11225	aldo/keto reductase	1.18

HD73_RS12105	nitrate reductase subunit alpha	1.17
HD73_RS12110	nitrate reductase subunit beta	1.15
HD73_RS05485	enoyl-CoA hydratase	1.14
HD73_RS08195	GNAT family N-acetyltransferase	1.13
HD73_RS28390	NADH-quinone oxidoreductase subunit NuoK	1.12
HD73_RS14010	F0F1 ATP synthase subunit alpha	1.1
HD73_RS12165	precorrin-2 dehydrogenase	1.09
HD73_RS09600	HD domain-containing protein	1.09
HD73_RS27120	NAD(P)/FAD-dependent oxidoreductase	1.08
HD73_RS27690	8-oxo-dGTP diphosphatase	1.08
HD73_RS01975	amidophosphoribosyltransferase	1.06
HD73_RS24335	MaoC/PaaZ C-terminal domain-containing protein	1.05
HD73_RS00365	hypoxanthine phosphoribosyltransferase	1.05
HD73_RS01955	phosphoribosylaminoimidazolesuccinocarboxamide synthase	1.04
HD73_RS24435	CBS domain-containing protein	1.04
HD73_RS26705	iron-sulfur cluster assembly scaffold protein SufU	1.03
HD73_RS09345	sulfurtransferase	1.01
HD73_RS18930	GNAT family N-acetyltransferase	1.01
HD73_RS28105	diguanylate cyclase	1.01
HD73_RS19445	long-chain fatty acid--CoA ligase	1
	**Energy metabolism**	
	**Downregulated genes**	
HD73_RS23230	4a-hydroxytetrahydrobiopterin dehydratase	-2.97
HD73_RS20765	uracil permease	-2.74
HD73_RS07185	cation-translocating P-type ATPase	-2.68
HD73_RS32925	HAD hydrolase family protein	-2.54
HD73_RS26575	HpcH/HpaI aldolase/citrate lyase family protein	-2.49
HD73_RS26580	phosphoribosyltransferase family protein	-1.97
HD73_RS25200	PepSY domain-containing protein	-1.47
HD73_RS02130	NADP-dependent succinate-semialdehyde dehydrogenase	-1.44
HD73_RS06020	IS21-like element IS232 family helper ATPase IstB	-1.43
HD73_RS21970	biotin synthase	-1.35
HD73_RS16815	collagen-like repeat preface domain-containing protein	-1.2
HD73_RS20725	orotate phosphoribosyltransferase	-1.2
HD73_RS15385	ABC transporter ATP-binding protein	-1.14
HD73_RS20745	carbamoyl-phosphate synthase large subunit	-1.13
HD73_RS20750	carbamoyl phosphate synthase small subunit	-1.09
HD73_RS16365	SagB/ThcOx family dehydrogenase	-1.07
HD73_RS13375	DMT family transporter	-1.04
HD73_RS20740	dihydroorotate oxidase B electron transfer subunit	-1.04

HD73_RS05395	NDxxF motif lipoprotein	-1.04
HD73_RS29180	superoxide dismutase [Mn]	-1.04
HD73_RS19035	nucleoside hydrolase	-1.03
	**Genetic information processing**	
	**Upregulated genes**	
HD73_RS19965	heavy metal translocating P-type ATPase	2.9
HD73_RS19970	copper chaperone CopZ	2.83
HD73_RS20790	isoleucine--tRNA ligase	1.87
HD73_RS20920	N-acetyltransferase	1.83
HD73_RS07330	tryptophan-tRNA ligase	1.63
HD73_RS20505	50S ribosomal protein L19	1.58
HD73_RS19955	HU family DNA-binding protein	1.58
HD73_RS20205	recombinase RecA	1.53
HD73_RS04355	replication-relaxation family protein	1.51
HD73_RS25135	leucine-tRNA ligase	1.49
HD73_RS29805	TnP I resolvase	1.46
HD73_RS29800	Tn3 family transposase	1.42
HD73_RS27330	glutaredoxin family protein	1.39
HD73_RS23470	D-tyrosyl-tRNA (Tyr) deacylase	1.37
HD73_RS29505	Tn3 family transposase	1.37
HD73_RS07570	ATP-dependent helicase	1.37
HD73_RS25155	tyrosine-type recombinase/integrase	1.33
HD73_RS17300	septum formation initiator family protein	1.29
HD73_RS06910	LexA family transcriptional regulator	1.26
HD73_RS06540	PH domain-containing protein	1.26
HD73_RS21015	cytochrome c oxidase assembly factor CtaG	1.23
HD73_RS23425	cysteine desulfurase	1.22
HD73_RS14590	signal peptidase I	1.21
HD73_RS16675	IS110 family transposase	1.18
HD73_RS24260	ThrS	1.17
HD73_RS23475	GTP diphosphokinase	1.17
HD73_RS00360	tRNA lysidine synthase	1.15
HD73_RS16945	RNaseH domain-containing protein	1.14
HD73_RS23970	thioredoxin	1.13
HD73_RS23420	tRNA 2-thiouridine (34) synthase MnmA	1.12
HD73_RS33440	ribosomal-processing cysteine protease Prp	1.11
HD73_RS00025	DNA gyrase subunit B	1.09
HD73_RS20905	16S rRNA (cytosine (1402)-N (4))-methyltransferase RsmH	1.09
HD73_RS20810	cell division protein SepF	1.09
HD73_RS26720	Fe-S cluster assembly ATPase SufC	1.08

HD73_RS21705	peptidylprolyl isomerase	1.07
HD73_RS04350	FtsK/SpoIIIE domain-containing protein	1.07
HD73_RS00015	S4 domain-containing protein YaaA	1.06
HD73_RS23635	GTPase ObgE	1.06
HD73_RS26715	Fe-S cluster assembly protein SufD	1.05
HD73_RS20375	YlxQ family RNA-binding protein	1.04
HD73_RS27265	SsrA-binding protein	1.04
HD73_RS26465	iron-sulfur cluster assembly accessory protein	1.02
HD73_RS21405	SDR family NAD(P)-dependent oxidoreductase	1.02
HD73_RS23085	ribosome silencing factor	1.02
HD73_RS22915	DNA repair protein RecO	1.01
HD73_RS19965	heavy metal translocating P-type ATPase	2.9
HD73_RS19970	copper chaperone CopZ	2.83
HD73_RS20790	isoleucine--tRNA ligase	1.87
HD73_RS20920	N-acetyltransferase	1.83
HD73_RS07330	tryptophan-tRNA ligase	1.63
HD73_RS20505	50S ribosomal protein L19	1.58
HD73_RS19955	HU family DNA-binding protein	1.58
HD73_RS20205	recombinase RecA	1.53
HD73_RS04355	replication-relaxation family protein	1.51
HD73_RS25135	leucine-tRNA ligase	1.49
HD73_RS29805	TnP I resolvase	1.46
HD73_RS29800	Tn3 family transposase	1.42
HD73_RS27330	glutaredoxin family protein	1.39
HD73_RS23470	D-tyrosyl-tRNA (Tyr) deacylase	1.37
HD73_RS29505	Tn3 family transposase	1.37
HD73_RS07570	ATP-dependent helicase	1.37
HD73_RS25155	tyrosine-type recombinase/integrase	1.33
HD73_RS17300	septum formation initiator family protein	1.29
HD73_RS06910	LexA family transcriptional regulator	1.26
HD73_RS06540	PH domain-containing protein	1.26
HD73_RS21015	cytochrome c oxidase assembly factor CtaG	1.23
HD73_RS23425	cysteine desulfurase	1.22
HD73_RS14590	signal peptidase I	1.21
HD73_RS16675	IS110 family transposase	1.18
HD73_RS24260	ThrS	1.17
HD73_RS23475	GTP diphosphokinase	1.17
HD73_RS00360	tRNA lysidine synthase	1.15
HD73_RS16945	RNaseH domain-containing protein	1.14
HD73_RS23970	thioredoxin	1.13
HD73_RS23420	tRNA 2-thiouridine (34) synthase MnmA	1.12

HD73_RS33440	ribosomal-processing cysteine protease Prp	1.11
HD73_RS00025	DNA gyrase subunit B	1.09
HD73_RS20905	16S rRNA (cytosine (1402)-N (4))-methyltransferase RsmH	1.09
HD73_RS20810	cell division protein SepF	1.09
HD73_RS26720	Fe-S cluster assembly ATPase SufC	1.08
HD73_RS21705	peptidylprolyl isomerase	1.07
HD73_RS04350	FtsK/SpoIIIE domain-containing protein	1.07
HD73_RS00015	S4 domain-containing protein YaaA	1.06
HD73_RS23635	GTPase ObgE	1.06
HD73_RS26715	Fe-S cluster assembly protein SufD	1.05
HD73_RS20375	YlxQ family RNA-binding protein	1.04
HD73_RS27265	SsrA-binding protein	1.04
	**Genetic information processing**	
	**Downregulated genes**	
HD73_RS26180	type IV secretory system conjugative DNA transfer family protein	-6.91
HD73_RS26440	site-specific integrase	-6.85
HD73_RS26155	FtsK/SpoIIIE domain-containing protein	-5.08
HD73_RS12845	chromosome-anchoring protein RacA	-4
HD73_RS07840	CalY family protein	-3.98
HD73_RS07835	Signal peptidase I	-2.49
HD73_RS16435	DinB family protein	-1.93
HD73_RS13210	HU family DNA-binding protein	-1.65
HD73_RS28680	IS4-like element IS231C family transposase	-1.57
HD73_RS19800	IS4-like element IS231C family transposase	-1.27
HD73_RS16395	Tn3 family transposase	-1.17
HD73_RS29490	Tn3 family transposase	-1.13
	**Signaling cellular processes**	
	**Upregulated genes**	
HD73_RS10645	branched-chain amino acid transport system II carrier protein	3.84
HD73_RS13160	OFA family MFS transporter	3.36
HD73_RS12500	sodium-dependent transporter	3.12
HD73_RS12505	alpha/beta hydrolase	2.86
HD73_RS08670	branched-chain amino acid transport system II carrier protein	2.86
HD73_RS03745	lactate permease LctP family transporter	2.85
HD73_RS07350	ABC transporter permease	2.81
HD73_RS07345	peptide ABC transporter substrate-binding protein	2.61
HD73_RS14955	MFS transporter	2.56

HD73_RS07355	ABC transporter permease	2.46
HD73_RS22500	multidrug efflux SMR transporter	2.41
HD73_RS28125	cell wall-binding protein EntA	2.24
HD73_RS15060	amino acid permease	2.21
HD73_RS01260	Amino acid transporter	2.2
HD73_RS08285	SseB family protein	2.14
HD73_RS23935	M15 family metallopeptidase	2.12
HD73_RS07360	ABC transporter ATP-binding protein	2.08
HD73_RS04720	SH3 domain-containing protein	2.03
HD73_RS19895	cell wall-binding protein EntD	2.03
HD73_RS18025	hemolysin family protein	1.85
HD73_RS07365	ATP-binding cassette domain-containing protein	1.82
HD73_RS15790	(2Fe-2S)-binding protein	1.8
HD73_RS04525	sodium:solute symporter	1.7
HD73_RS18420	amino acid permease	1.7
HD73_RS12755	ABC transporter permease subunit	1.66
HD73_RS19935	GABA permease	1.6
HD73_RS05065	cell wall-binding protein EntC	1.55
HD73_RS08615	sodium-dependent transporter	1.53
HD73_RS15735	dipeptide epimerase	1.51
HD73_RS15765	DedA family protein	1.51
HD73_RS05345	ABC transporter ATP-binding protein/permease	1.5
HD73_RS05175	amino acid permease	1.5
HD73_RS28650	(Fe-S)-binding protein	1.49
HD73_RS15740	C40 family peptidase	1.48
HD73_RS15070	M20 family metallopeptidase	1.47
HD73_RS15795	(2Fe-2S)-binding protein	1.45
HD73_RS03560	2-hydroxycarboxylate transporter family protein	1.44
HD73_RS22505	DUF3975 family protein	1.44
HD73_RS29510	TnP I resolvase	1.41
HD73_RS01035	molybdate ABC transporter substrate-binding protein	1.4
HD73_RS14890	collagenase ColA	1.39
HD73_RS03215	aromatic acid exporter family protein	1.39
HD73_RS08625	glutamate/aspartate:proton symporter GltP	1.37
HD73_RS26730	methionine ABC transporter substrate-binding lipoprotein MetQ	1.37
HD73_RS01030	molybdenum ABC transporter permease	1.36
HD73_RS02875	TerD family protein	1.34
HD73_RS22475	rhodanese-like domain-containing protein	1.34
HD73_RS11075	peptidase	1.34
HD73_RS15685	Xaa-Pro dipeptidyl-peptidase	1.32

HD73_RS12760	ABC transporter permease subunit	1.32
HD73_RS27225	siderophore ABC transporter substrate-binding protein	1.3
HD73_RS06170	heavy metal-binding domain-containing protein	1.29
HD73_RS15050	fatty acid desaturase	1.28
HD73_RS21140	GapA-binding peptide SR1P	1.27
HD73_RS26070	biotin transporter BioY	1.27
HD73_RS25220	gamma carbonic anhydrase family protein	1.26
HD73_RS09595	RNA chaperone Hfq	1.26
HD73_RS29035	carbon starvation protein CstA	1.24
HD73_RS08250	ABC transporter ATP-binding protein	1.24
HD73_RS06680	beta-channel forming cytolysin CytK2	1.23
HD73_RS03770	peptide MFS transporter	1.23
HD73_RS29915	ParM/StbA family protein	1.21
HD73_RS04400	3D domain-containing protein	1.21
HD73_RS11985	oxalate:formate antiporter	1.21
HD73_RS05560	ABC transporter ATP-binding protein	1.2
HD73_RS29020	pyridoxine/pyridoxal/pyridoxamine kinase	1.18
HD73_RS18410	hypothetical protein	1.18
HD73_RS21375	maltosaccharide ABC transporter permease MalD	1.17
HD73_RS07985	formate/nitrite transporter family protein	1.16
HD73_RS21630	phosphocarrier protein HPr	1.15
HD73_RS10500	Nramp family divalent metal transporter	1.15
HD73_RS20820	peptidoglycan editing factor PgeF	1.14
HD73_RS26725	methionine ABC transporter substrate-binding lipoprotein MetQ	1.14
HD73_RS03985	MFS transporter	1.14
HD73_RS15205	cell wall-binding protein EntB	1.13
HD73_RS08730	inorganic phosphate transporter family protein	1.11
HD73_RS29030	YbdD/YjiX family protein	1.1
HD73_RS23940	acyl-CoA thioesterase	1.09
HD73_RS20700	Xaa-Pro peptidase family protein	1.07
HD73_RS09110	zinc metallopeptidase	1.07
HD73_RS00755	energy-coupling factor ABC transporter	1.06
HD73_RS08620	M23 family metallopeptidase	1.06
HD73_RS20265	BMP family ABC transporter substrate-binding protein	1.04
HD73_RS27055	ferritin	1.03
HD73_RS09530	cation:proton antiporter	1.02
HD73_RS15045	immune inhibitor A	1.01
HD73_RS09325	dynamin family protein	1.01
	**Signaling cellular processes**	
	**Downregulated genes**	

HD73_RS26425	AimR family lysis-lysogeny pheromone receptor	-7.13
HD73_RS07850	biofilm matrix protein CalY	-4.63
HD73_RS26985	M4 family metallopeptidase	-3.03
HD73_RS09395	CpsD/CapB family tyrosine-protein kinase	-2.75
HD73_RS11865	WXG100 family type VII secretion target	-2.74
HD73_RS09385	acyltransferase	-2.6
HD73_RS09390	Wzz/FepE/Etk N-terminal domain-containing protein	-2.41
HD73_RS04580	efflux RND transporter permease subunit	-2.27
HD73_RS09400	sugar transferase	-1.99
HD73_RS09405	oligosaccharide flippase family protein	-1.98
HD73_RS11630	HNH endonuclease	-1.94
HD73_RS09415	glycosyltransferase	-1.91
HD73_RS02135	multidrug efflux SMR transporter	-1.8
HD73_RS11910	DUF4176 domain-containing protein	-1.75
HD73_RS17205	LPXTG cell wall anchor domain-containing protein	-1.71
HD73_RS03675	M4 family metallopeptidase	-1.53
HD73_RS31600	MFS transporter	-1.51
HD73_RS09420	glycosyltransferase family 4 protein	-1.5
HD73_RS24770	SpaH/EbpB family LPXTG-anchored major pilin	-1.5
HD73_RS11870	type VII secretion protein EsaA	-1.4
HD73_RS07210	ammonium transporter	-1.36
HD73_RS11885	type VII secretion protein EssB	-1.36
HD73_RS09515	sodium/solute symporter	-1.35
HD73_RS05605	WXG100 family type VII secretion target	-1.33
HD73_RS09410	O-antigen ligase family protein	-1.25
HD73_RS02640	aromatic acid/H+ symport family MFS transporter	-1.22
HD73_RS11890	type VII secretion protein EssC	-1.21
HD73_RS09520	aspartate aminotransferase family protein	-1.21
HD73_RS21840	FixH family protein	-1.09
HD73_RS15640	M3 family metallopeptidase	-1.08
HD73_RS13770	hypothetical protein	-1.05
HD73_RS08400	dicarboxylate/amino acid:cation symporter	-1.04
HD73_RS12650	DegV family protein	-1.02
HD73_RS13775	TSUP family transporter	-1.01
	**Sporulation**	
	**Upregulated genes**	
HD73_RS21355	sporulation sensor histidine kinase KinB	1.19
HD73_RS14455	Sporulation kinase C	1.43
	**Sporulation**	
	**Downregulated genes**	
HD73_RS21775	anti-sigma F factor antagonist	-3.12

HD73_RS21770	anti-sigma F factor	-2.53
HD73_RS21765	RNA polymerase sporulation sigma factor SigF	-2.53
HD73_RS06865	aspartyl-phosphate phosphatase Spo0E family protein	-2.09
HD73_RS23120	sporulation histidine kinase inhibitor Sda	-1.55
HD73_RS00355	stage II sporulation protein E	-1.52
HD73_RS20835	RNA polymerase sporulation sigma factor SigE	-1.5
HD73_RS21745	stage V sporulation protein SpoVAB	-1.31
HD73_RS19765	sporulation inhibitor of replication protein SirA	-1.19
HD73_RS30725	RapH N-terminal domain-containing protein	-1.14
HD73_RS06260	Response regulator aspartate phosphatase inhibitor 1	-3.18
HD73_RS33570	Response regulator aspartate phosphatase inhibitor 8	-1.2
	**Transcriptional regulators**	
	**Upregulated genes**	
HD73_RS22490	TetR/AcrR family transcriptional regulator	2.58
HD73_RS04090	helix-turn-helix transcriptional regulator	2.27
HD73_RS04095	helix-turn-helix domain-containing protein	2.04
HD73_RS03650	metalloregulator ArsR/SmtB family transcription factor	1.89
HD73_RS18030	MerR family transcriptional regulator	1.84
HD73_RS08390	MarR family transcriptional regulator	1.63
HD73_RS21650	GntR family transcriptional regulator	1.6
HD73_RS22645	helix-turn-helix domain-containing protein	1.52
HD73_RS19975	metal-sensing transcriptional repressor	1.51
HD73_RS30215	helix-turn-helix transcriptional regulator	1.51
HD73_RS26780	PTS sugar transporter subunit IIC	1.48
HD73_RS24695	RNA polymerase sigma factor	1.45
HD73_RS06360	HTH-type transcriptional regulator Hpr	1.37
HD73_RS04360	helix-turn-helix domain-containing protein	1.36
HD73_RS24210	DNA binding protein	1.3
HD73_RS08015	poly-beta-hydroxybutyrate-responsive repressor	1.28
HD73_RS19820	transcriptional repressor LexA	1.27
HD73_RS23430	Rrf2 family transcriptional regulator	1.24
HD73_RS16230	TetR/AcrR family transcriptional regulator	1.23
HD73_RS04085	helix-turn-helix domain-containing protein	1.21
HD73_RS01485	helix-turn-helix domain-containing protein	1.18
HD73_RS20585	transcription factor FapR	1.13
HD73_RS11110	ArsR family transcriptional regulator	1.13
HD73_RS27840	peptide chain release factor 2	1.11
HD73_RS15065	IclR family transcriptional regulator	1.09
HD73_RS04440	DNA-binding protein	1.04
	**Transcriptional regulators**	
	**Downregulated genes**	

HD73_RS03760	ArsR family transcription factor	-8.03
HD73_RS26170	helix-turn-helix transcriptional regulator	-7.63
HD73_RS26420	helix-turn-helix domain-containing protein	-5.71
HD73_RS26410	helix-turn-helix domain-containing protein	-5.47
HD73_RS13890	metalloregulator ArsR/SmtB family transcription factor	-2.51
HD73_RS20770	bifunctional pyrimidine operon transcriptional regulator	-2.31
HD73_RS20840	sigma-E processing peptidase SpoIIGA	-2.12
HD73_RS06730	response regulator transcription factor	-1.29
HD73_RS24645	forespore capture DNA-binding protein RefZ	-1.08
HD73_RS10670	LysR family transcriptional regulator	-1.05
	**Virulence**	
	**Upregulated genes**	
HD73_RS16355	M4 family metallopeptidase	3.97
HD73_RS14445	alpha-helical pore-forming toxin family protein	3.16
HD73_RS14440	hemolytic enterotoxin HBL lytic component L1	3.01
HD73_RS14435	hypothetical protein	2.89
HD73_RS04025	M6 family metalloprotease immune inhibitor InhA2	2.88
HD73_RS04240	phage major capsid protein	2.85
HD73_RS04070	sphingomyelinase C	2.66
HD73_RS18145	M60 family metallopeptidase	2.64
HD73_RS04065	phospholipase CerA	2.62
HD73_RS18140	M4 family metallopeptidase	2.6
HD73_RS04320	hypothetical protein	2.59
HD73_RS16960	DUF3937 family protein	2.54
HD73_RS10530	non-hemolytic enterotoxin NHE subunit A	2.43
HD73_RS20115	phosphatidylinositol diacylglycerol-lyase	2.4
HD73_RS14450	hemolytic enterotoxin HBL binding subunit HblA	2.39
HD73_RS04215	HNH endonuclease	2.35
HD73_RS00910	glucosamine--fructose-6-phosphate aminotransferase	2.34
HD73_RS01655	hypothetical protein	2.3
HD73_RS04205	hypothetical protein	2.29
HD73_RS04325	hypothetical protein	2.28
HD73_RS27200	anthrolysin O	2.26
HD73_RS04310	tail fiber domain-containing protein	2.26
HD73_RS10540	non-hemolytic enterotoxin NHE subunit C	2.2
HD73_RS30200	S-layer homology domain-containing protein	2.19
HD73_RS10535	non-hemolytic enterotoxin NHE subunit B	2.16
HD73_RS01675	tail protein	2.14
HD73_RS04210	hypothetical protein	2.06
HD73_RS28705	M4 family metallopeptidase	2.06
HD73_RS11295	peptide ABC transporter substrate-binding protein	2.05

HD73_RS04315	site-specific integrase	2.02
HD73_RS20120	peptidase	2.02
HD73_RS11645	phage portal protein	2
HD73_RS04220	phage terminase small subunit P27 family	1.96
HD73_RS04235	Clp protease ClpP	1.91
HD73_RS19210	peptide ABC transporter substrate-binding protein	1.9
HD73_RS04230	phage portal protein	1.89
HD73_RS01660	phage head closure protein	1.88
HD73_RS01650	phage major capsid protein	1.87
HD73_RS04100	phage regulatory protein	1.86
HD73_RS04195	hypothetical protein	1.85
HD73_RS29625	S-layer homology domain-containing protein	1.83
HD73_RS04225	terminase large subunit	1.82
HD73_RS11665	phage head closure protein	1.81
HD73_RS04185	hypothetical protein	1.8
HD73_RS04200	hypothetical protein	1.8
HD73_RS10055	PH domain-containing protein	1.77
HD73_RS04305	phage tail family protein	1.75
HD73_RS11660	head-tail connector protein	1.67
HD73_RS04245	head-tail connector protein	1.66
HD73_RS24205	CPBP family intramembrane metalloprotease	1.62
HD73_RS18905	hypothetical protein	1.61
HD73_RS19220	peptide ABC transporter substrate-binding protein	1.56
HD73_RS26255	phage head closure protein	1.54
HD73_RS11640	terminase large subunit	1.51
HD73_RS10545	LysE family translocator	1.47
HD73_RS01645	Clp protease ClpP	1.44
HD73_RS32640	S-layer homology domain-containing protein	1.4
HD73_RS20045	bifunctional diguanylate cyclase/phosphodiesterase	1.4
HD73_RS04300	phage tail tape measure protein	1.39
HD73_RS08705	NO-inducible flavohemoprotein	1.38
HD73_RS04270	hypothetical protein	1.38
HD73_RS26260	head-tail connector protein	1.36
HD73_RS18150	methyl-accepting chemotaxis protein	1.28
HD73_RS15745	peptide ABC transporter substrate-binding protein	1.24
HD73_RS23910	ABC transporter permease	1.24
HD73_RS13915	alpha-helical pore-forming toxin family protein	1.23
HD73_RS01665	HK97 gp10 family phage protein	1.2
HD73_RS01640	phage portal protein	1.19
HD73_RS03440	collagenase ColA	1.13
HD73_RS27430	phage head closure protein	1.11

HD73_RS13905	HBL/NHE enterotoxin family protein	1.06
HD73_RS13910	HBL/NHE enterotoxin family protein	1.03
HD73_RS01670	DUF3168 domain-containing protein	1.02
HD73_RS11720	phage tail family protein	1.01
HD73_RS10855	enterotoxin EntFM	1
	**Virulence**	
	**Downregulated genes**	
HD73_RS26250	HK97 gp10 family phage protein	-8.29
HD73_RS26225	phage tail family protein	-7.7
HD73_RS26240	tail protein	-7.61
HD73_RS26150	replication-relaxation family protein	-7.33
HD73_RS26210	phage holin family protein	-6.31
HD73_RS26245	DUF3168 domain-containing protein	-5.63
HD73_RS26220	tail fiber domain-containing protein	-3.6
HD73_RS33860	hypothetical protein	-2.9
HD73_RS02805	glycosyl hydrolase family 18 protein	-2.43
HD73_RS12475	Bacillolysin	-2.08
HD73_RS15855	lytic polysaccharide monooxygenase	-1.99
HD73_RS26310	hypothetical protein	-1.87
HD73_RS26315	hypothetical protein	-1.75
HD73_RS26305	hypothetical protein	-1.75
HD73_RS16640	glycosyl hydrolase family 8	-1.72
HD73_RS26300	hypothetical protein	-1.57
HD73_RS11105	serine protease	-1.54
HD73_RS26325	hypothetical protein	-1.52
HD73_RS26285	phage terminase small subunit P27 family	-1.48
HD73_RS26290	HNH endonuclease	-1.46
HD73_RS18900	VaFE repeat-containing surface-anchored protein	-1.29
HD73_RS16875	cysteine dioxygenase family protein	-1.21
HD73_RS11655	phage major capsid protein	-1.15
HD73_RS26265	phage major capsid protein	-1.15
HD73_RS26320	hypothetical protein	-1.12
HD73_RS03695	VanW family protein	-1.1
HD73_RS08640	hypothetical protein	-1.04
HD73_RS14015	2TM domain-containing protein	-1.01
	**Others and unknown function**	
	**Upregulated genes**	
HD73_RS16990	DUF3914 domain-containing protein	4.02
HD73_RS33100	hypothetical protein	3.08
HD73_RS15780	YxcD family protein	2.79
HD73_RS15775	hypothetical protein	2.58

HD73_RS30190	hypothetical protein	2.53
HD73_RS13655	hypothetical protein	2.46
HD73_RS30195	hypothetical protein	2.4
HD73_RS28755	hypothetical protein	2.26
HD73_RS04715	hypothetical protein	2.18
HD73_RS24160	hypothetical protein	2.11
HD73_RS28940	DUF3817 domain-containing protein	2.1
HD73_RS04520	DUF3311 domain-containing protein	1.92
HD73_RS11685	hypothetical protein	1.81
HD73_RS24700	hypothetical protein	1.71
HD73_RS27115	DUF1641 domain-containing protein	1.69
HD73_RS06235	hypothetical protein	1.68
HD73_RS22255	DUF2627 domain-containing protein	1.68
HD73_RS12375	DUF4238 domain-containing protein	1.68
HD73_RS24690	hypothetical protein	1.65
HD73_RS10600	DUF896 domain-containing protein	1.64
HD73_RS08735	DUF47 domain-containing protein	1.62
HD73_RS21585	hypothetical protein	1.59
HD73_RS30205	hypothetical protein	1.59
HD73_RS29935	hypothetical protein	1.58
HD73_RS26900	DUF3938 domain-containing protein	1.55
HD73_RS21110	hypothetical protein	1.54
HD73_RS15600	hypothetical protein	1.54
HD73_RS27410	hypothetical protein	1.54
HD73_RS11080	DUF4083 domain-containing protein	1.5
HD73_RS22585	hypothetical protein	1.47
HD73_RS05000	hypothetical protein	1.46
HD73_RS04335	hypothetical protein	1.46
HD73_RS15555	hypothetical protein	1.4
HD73_RS19315	HesB/YadR/YfhF family protein	1.36
HD73_RS26630	hypothetical protein	1.36
HD73_RS29930	hypothetical protein	1.35
HD73_RS30765	hypothetical protein	1.35
HD73_RS04380	hypothetical protein	1.31
HD73_RS18455	YojF family protein	1.31
HD73_RS19930	hypothetical protein	1.26
HD73_RS04705	DUF3965 domain-containing protein	1.25
HD73_RS11390	hypothetical protein	1.23
HD73_RS03265	YgzB family protein	1.22
HD73_RS30220	hypothetical protein	1.22
HD73_RS12805	YdcF family protein	1.2

HD73_RS25010	hypothetical protein	1.18
HD73_RS22685	DUF3966 domain-containing protein	1.18
HD73_RS08245	hypothetical protein	1.16
HD73_RS20970	hypothetical protein	1.15
HD73_RS11545	DUF3954 domain-containing protein	1.15
HD73_RS29830	hypothetical protein	1.14
HD73_RS07265	hypothetical protein	1.11
HD73_RS07830	hypothetical protein	1.11
HD73_RS05005	hypothetical protein	1.1
HD73_RS23960	YslB family protein	1.09
HD73_RS21060	YlaN family protein	1.07
HD73_RS30770	hypothetical protein	1.07
HD73_RS07580	YjcG family protein	1.06
HD73_RS23535	DUF2905 domain-containing protein	1.06
HD73_RS09075	YpiF family protein	1.05
HD73_RS20685	YicC family protein	1.05
HD73_RS26375	DUF3954 domain-containing protein	1.05
HD73_RS26610	DUF86 domain-containing protein	1.04
HD73_RS03205	hypothetical protein	1.04
HD73_RS09005	hypothetical protein	1.04
HD73_RS10440	DUF554 domain-containing protein	1.03
HD73_RS07335	DUF3899 domain-containing protein	1.03
HD73_RS22670	hypothetical protein	1.02
HD73_RS14555	YjiH family protein	1.02
HD73_RS22815	DUF1189 domain-containing protein	1.02
HD73_RS02895	YceG family protein	1.02
HD73_RS21620	DUF3915 domain-containing protein	1.01
HD73_RS28130	DUF3910 family protein	1.01
HD73_RS26100	YuiB family protein	1
	**Others and unknown function**	
	**Downregulated genes**	
HD73_RS26175	hypothetical protein	-7.95
HD73_RS26185	hypothetical protein	-7.74
HD73_RS26160	hypothetical protein	-6.93
HD73_RS26165	hypothetical protein	-6.84
HD73_RS26145	hypothetical protein	-6.78
HD73_RS26415	hypothetical protein	-4.79
HD73_RS07190	hypothetical protein	-3.91
HD73_RS07195	hypothetical protein	-3.75
HD73_RS17375	hypothetical protein	-3.02
HD73_RS30730	hypothetical protein	-3.02

HD73_RS07180	YtxH domain-containing protein	-2.65
HD73_RS07200	hypothetical protein	-2.62
HD73_RS21260	DUF3993 domain-containing protein	-2.59
HD73_RS30735	hypothetical protein	-2.59
HD73_RS21350	DUF3967 domain-containing protein	-2.36
HD73_RS12645	DUF2535 family protein	-2.16
HD73_RS25400	DUF3953 domain-containing protein	-2.15
HD73_RS12305	hypothetical protein	-2.1
HD73_RS25390	YtzI protein	-2.08
HD73_RS09245	hypothetical protein	-2.06
HD73_RS16925	hypothetical protein	-1.96
HD73_RS11605	hypothetical protein	-1.91
HD73_RS13885	DUF3221 domain-containing protein	-1.85
HD73_RS33185	hypothetical protein	-1.65
HD73_RS11555	hypothetical protein	-1.59
HD73_RS24810	DUF4288 domain-containing protein	-1.59
HD73_RS04140	hypothetical protein	-1.58
HD73_RS26365	hypothetical protein	-1.57
HD73_RS08675	hypothetical protein	-1.57
HD73_RS18010	DUF3923 family protein	-1.56
HD73_RS14360	hypothetical protein	-1.56
HD73_RS11610	hypothetical protein	-1.54
HD73_RS18005	hypothetical protein	-1.49
HD73_RS18465	hypothetical protein	-1.47
HD73_RS13120	hypothetical protein	-1.46
HD73_RS24430	DUF3949 domain-containing protein	-1.39
HD73_RS25405	YczI family protein	-1.35
HD73_RS20030	hypothetical protein	-1.33
HD73_RS13835	hypothetical protein	-1.3
HD73_RS18910	hypothetical protein	-1.3
HD73_RS13590	hypothetical protein	-1.28
HD73_RS24805	DUF3949 domain-containing protein	-1.27
HD73_RS31810	hypothetical protein	-1.25
HD73_RS33365	hypothetical protein	-1.25
HD73_RS14990	DUF2812 domain-containing protein	-1.25
HD73_RS01065	YrzO family protein	-1.22
HD73_RS11600	hypothetical protein	-1.18
HD73_RS12215	hypothetical protein	-1.16
HD73_RS06485	DUF6359 domain-containing protein	-1.16
HD73_RS15915	hypothetical protein	-1.16
HD73_RS18385	hypothetical protein	-1.15

HD73_RS07775	DUF4878 domain-containing protein	-1.13
HD73_RS16375	hypothetical protein	-1.11
HD73_RS12220	hypothetical protein	-1.1
HD73_RS11595	hypothetical protein	-1.09
HD73_RS33490	hypothetical protein	-1.08
HD73_RS13740	hypothetical protein	-1.07
HD73_RS29780	hypothetical protein	-1.06
HD73_RS33770	hypothetical protein	-1.06
HD73_RS21360	hypothetical protein	-1.05
HD73_RS31210	hypothetical protein	-1.05
HD73_RS14320	hypothetical protein	-1.04
HD73_RS29700	hypothetical protein	-1.03
HD73_RS26745	hypothetical protein	-1.03
HD73_RS33295	hypothetical protein	-1.02
HD73_RS11130	DUF805 domain-containing protein	-1.01
HD73_RS04850	hypothetical protein	-1.01

SUPPLEMENTARY TABLE 3 RNA-seq for genes of relevance that affect Spo0A activity at T_0 in Δ*cdsR*.

No.	Gene ID	Gene name	Annotation	Log_2(foldchange)
1	HD73_RS16865	*kinA*	Sporulation kinase A	NS[a]
2	HD73_RS21355	*kinB*	Sporulation kinase B	1.19
3	HD73_RS14455	*kinC*	Sporulation kinase C	1.43
4	HD73_RS08125	*kinD*	Sporulation kinase D	NS
5	HD73_RS16510	*kinE*	Sporulation sensor kinase E	NS
6	HD73_RS28605	*spo0F*	Sporulation initiation phosphotransferase F	NS
7	HD73_RS23640	*spo0B*	Sporulation initiation phosphotransferase B	NS
8	HD73_RS22275	*spo0A*	Stage 0 sporulation protein A	NS
9	HD73_RS23120	*sda*	Sporulation inhibitor sda	-1.55
10	HD73_RS06260	*phr1*	Response regulator aspartate phosphatase inhibitor 1	-3.18
11	HD73_RS06255	*rap1*	Response regulator aspartate phosphatase 1	NS
12	HD73_RS12280	*phr2*	Response regulator aspartate phosphatase inhibitor 2	NS
13	HD73_RS12275	*rap2*	Response regulator aspartate phosphatase 2	NS
14	HD73_RS33955	*phr3*	Response regulator aspartate phosphatase inhibitor 3	NS
15	HD73_RS18655	*rap3*	Response regulator aspartate phosphatase 3	NS
16	HD73_RS33410	*phr4*	Response regulator aspartate phosphatase inhibitor 4	NS
17	HD73_RS18810	*rap4*	Response regulator aspartate phosphatase 4	NS
18	HD73_RS27360	*phr5*	Response regulator aspartate phosphatase inhibitor 5	NS
19	HD73_RS27365	*rap5*	Response regulator aspartate phosphatase 5	NS
20	HD73_RS34015	*phr6*	Response regulator aspartate phosphatase inhibitor 6	NS
21	HD73_RS30515	*rap6*	Response regulator aspartate phosphatase 6	NS
22	HD73_RS32545	*phr7*	Response regulator aspartate	NS

			phosphatase inhibitor 7	
23	HD73_RS30065	*rap7*	Response regulator aspartate phosphatase 7	NS
24	HD73_RS33570	*phr8*	Response regulator aspartate phosphatase inhibitor 8	-1.2
25	HD73_RS30725	*rap8*	Response regulator aspartate phosphatase 8	-1.14
26	HD73_RS29280	*spo0E*	Aspartyl-phosphate phosphatas Spo0E	NS
27	HD73_RS08120	*ynzD*	Stage 0 sporulation regulatory protein	NS
28	HD73_RS06865	*yisI*	Stage 0 sporulation regulatory protein	-2.09
29	HD73_RS13390	*spo0E2*	Stage 0 sporulation regulatory protein	NS
30	HD73_RS32920	*spo0E3*	Stage 0 sporulation regulatory protein	NS
31	HD73_RS09590	*spo0E4*	Stage 0 sporulation regulatory protein	NS

[a]NS indicates no significance.

SUPPLEMENTARY TABLE 4 List of species in Figure S4

Species	Accession	Cover	E-value	Identity
Bacillus thuringiensis	WP_000081428.1	100%	5E-51	100%
Bacillus toyonensis	AHA09182.1	100%	5E-51	100%
Bacillus tropicus	WP_001075218.1	100%	1E-48	100%
Bacillus albus	WP_071757664.1	94%	1E-15	56%
Bacillus luti	WP_151624709.1	95%	1E-12	44%
Bacillus nitratireducens	QUG82547.1	100%	3E-65	96%
Bacillus cereus	WP_000081428.1	100%	5E-51	100%
Bacillus anthracis	AAP24629.1	100%	1E-66	99%
Bacillus cytotoxicus	ABS20880.1	100%	4E-64	95%
Bacillus pseudomycoides	AIK38417.1	100%	2E-63	94%
Bacillus manliponensis	WP_034641753.1	100%	5E-47	93%
Bacillus clarus	WP_042981873.1	100%	8E-47	94%
Bacillus fungorum	WP_098760748.1	88%	1E-19	62%
Bacillus paramycoides	WP_071719892.1	88%	1E-18	60%
Bacillus pacificus	UEP94152.1	100%	1E-66	99%
Paenibacillus fonticola	WP_019637278.1	87%	1E-14	54%
Selenomonas infelix	WP_006692051.1	68%	3E-15	48%
Selenomonas sp. Oral taxon 126	WP_066849122.1	68%	3E-15	48%
Paenibacillus segetis	WP_188535426.1	69%	3E-14	50%
Paenibacillus sanguinis	WP_018752818.1	68%	4E-14	54%
Selenomonas sp. Oral taxon 892	WP_021685476.1	68%	2E-14	46%
Paenibacillus anaericanus	WP_127192099.1	69%	2E-14	50%
Selenomonas massiliensis	WP_106627966.1	68%	2E-14	46%
Paenibacillus lupini	WP_167280536.1	69%	3E-14	48%
Selenomonadaceae bacterium	MBQ9615200.1	69%	1E-14	47%
Paenibacillus woosongensis	WP_155611636.1	66%	7E-14	51%
Clostridium frigidicarnis	WP_090038875.1	68%	3E-13	49%
Clostridium sp. P21	WP_169300372.1	69%	1E-14	47%
Paenibacillus montanisoli	WP_112882076.1	69%	3E-14	50%
Clostridium niameyense	WP_050608290.1	96%	7E-22	43%
Paenibacillus rubinfantis	WP_059042914.1	87%	7E-22	54%
Paenibacillus	WP_155605898.1	87%	7E-22	55%
Paenibacillus sp. TCA20	WP_047911403.1	90%	7E-22	49%
Paenibacillus oralis	WP_128633703.1	95%	1E-21	51%
Paenibacillus macerans	WP_036623091.1	95%	1E-21	51%
Clostridium zeae	WP_206872424.1	86%	1E-21	48%

Paenibacillus nanensis	WP_119602043.1	87%	1E-21	52%
Paenibacillaceae bacterium	MBW4838226.1	87%	2E-26	54%
Paenibacillus sp. PDC88	WP_090725784.1	90%	2E-21	48%
Paenibacillus alvei	WP_005545657.1	87%	3E-21	49%
Bacillus mobilis	WP_088029454.1	86%	5E-21	48%
Clostridium carboxidivorans	WP_179945238.1	86%	5E-21	46%
Paenibacillus lentus	WP_125081358.1	88%	5E-21	49%
Clostridium sp. YIM B02565	WP_202769407.1	86%	6E-21	46%
Paenibacillus barengoltzii	WP_085278574.1	95%	6E-21	51%
Paenibacillus spiritus	WP_150458581.1	82%	7E-21	51%
Paenibacillus cellulosilyticus	WP_110044775.1	85%	9E-21	46%
Paenibacillus bouchesdurhonensis	WP_110930040.1	87%	9E-21	51%
Clostridium estertheticum	WP_216191970.1	86%	1E-20	49%
Paenibacillus glacialis	WP_068531518.1	97%	3E-20	44%
Paenibacillus xerothermodurans	WP_089199760.1	86%	3E-20	50%
Paenibacillus harenae	WP_028610712.1	87%	3E-20	49%
Paenibacillus kobensis	WP_127533857.1	85%	3E-20	51%
Paenibacillus turicensis	WP_210088069.1	87%	4E-20	49%
Paenibacillus massiliensis	WP_018887203.1	87%	4E-20	49%
Paenibacillus lutimineralis	WP_126998228.1	84%	5E-20	49%
Paenibacillus sp. SDF0028	WP_142546644.1	87%	9E-20	47%
Paenibacillus phocaensis	WP_068785631.1	95%	1E-19	48%
Clostridium sulfidigenes	WP_051824141.1	90%	1E-19	42%
Selenomonas ruminantium	WP_029546300.1	85%	1E-24	48%
Paenibacillus sp. P22	CDN44879.1	83%	2E-19	51%
Paenibacillus popilliae	WP_006284165.1	92%	2E-19	45%
Clostridium magnum DSM 2767	KZL91388.1	83%	2E-19	44%
Clostridium cavendishii	WP_072990280.1	88%	2E-19	46%
Paenibacillus aquistagni	WP_085495680.1	86%	2E-19	48%
Clostridium psychrophilum	WP_216286715.1	86%	2E-19	48%
Paenibacillus yonginensis	WP_068695989.1	88%	4E-18	46%
Paenibacillus bovis	WP_060535254.1	88%	8E-18	44%
Paenibacillus ihuae	WP_054940683.1	79%	1E-22	43%
Paenibacillus stellifer	WP_038696199.1	79%	1E-22	43%
Paenibacillus pinistramenti	WP_138492986.1	87%	2E-17	45%
Paenibacillus wulumuqiensis	WP_046214047.1	88%	7E-17	43%
Paenibacillus physcomitrellae	WP_094093349.1	88%	2E-15	43%
Paenibacillus agaridevorans	WP_214629451.1	84%	5E-15	45%

SUPPLEMENTARY TABLE S5 Analysis of the Δ*cdsR* mutant transcriptome data for *lrgA* homologs

Gene ID	Gene name	HD73_FPKM	Δ*cdsR*_FPKM	Log_2(foldchange)
HD73_RS29150	*lrgA*	278.7111	1426.156	2.36
HD73_RS19705	*cidA*	19.17225	10.99018	NS[a]
HD73_RS20090	*clhA1*	45.86919	26.2142	NS
HD73_RS27290	*clhA2*	54.46333	26.99775	NS

[a]NS indicates no significance.
